# Interplay of intracellular and trans‐cellular DNA methylation in natural archaeal consortia

**DOI:** 10.1111/1758-2229.13258

**Published:** 2024-04-08

**Authors:** Oleg N. Reva, Violetta La Cono, Francesca Crisafi, Francesco Smedile, Manasi Mudaliyar, Debnath Ghosal, Laura Giuliano, Mart Krupovic, Michail M. Yakimov

**Affiliations:** ^1^ Department of Biochemistry, Genetics and Microbiology, Centre for Bioinformatics and Computational Biology University of Pretoria Pretoria South Africa; ^2^ Extreme Microbiology, Biotechnology and Astrobiology Group Institute of Polar Sciences, ISP‐CNR Messina Italy; ^3^ Department of Biochemistry and Pharmacology, Bio21 Molecular Science and Biotechnology Institute The University of Melbourne Melbourne Victoria Australia; ^4^ ARC Centre for Cryo‐electron Microscopy of Membrane Proteins, Bio21 Molecular Science and Biotechnology Institute University of Melbourne Parkville Victoria Australia; ^5^ Mediterranean Science Commission (CIESM) Monaco; ^6^ Istitut Pasteur, Archaeal Virology Unit Université Paris Cité Paris France

## Abstract

DNA methylation serves a variety of functions across all life domains. In this study, we investigated archaeal methylomics within a tripartite xylanolytic halophilic consortium. This consortium includes *Haloferax lucertense* SVX82, *Halorhabdus* sp. SVX81, and an ectosymbiotic *Candidatus* Nanohalococcus occultus SVXNc, a nano‐sized archaeon from the DPANN superphylum. We utilized PacBio SMRT and Illumina cDNA sequencing to analyse samples from consortia of different compositions for methylomics and transcriptomics. Endogenous *c*TAG methylation, typical of *Haloferax*, was accompanied in this strain by methylation at four other motifs, including GDG*c*HC methylation, which is specific to the ectosymbiont. Our analysis of the distribution of methylated and unmethylated motifs suggests that autochthonous cTAG methylation may influence gene regulation. The frequency of GRAGA*a*G methylation increased in highly expressed genes, while C*c*TTG and GTCG*a*GG methylation could be linked to restriction‐modification (RM) activity. Generally, the RM activity might have been reduced during the evolution of this archaeon to balance the protection of cells from intruders, the reduction of DNA damage due to self‐restriction in stressful environments, and the benefits of DNA exchange under extreme conditions. Our methylomics, transcriptomics and complementary electron cryotomography (cryo‐ET) data suggest that the nanohaloarchaeon exports its methyltransferase to methylate the *Haloferax* genome, unveiling a new aspect of the interaction between the symbiont and its host.

## Graphical abstract

This study examined genome methylation in *Haloferax lucertense* and its DPANN symbiont, *Candidatus* Nanohalococcus occultus, within an archaeal halophilic xylan‐degrading community. It revealed a complex methylation pattern in the genome of *H. lucertense* and presented the first evidence of cross‐methylation by the symbiont's methyltransferases. Statistical analysis indicated a non‐random distribution of methylated canonical motifs and a correlation between genome methylation, growth conditions, and community composition.

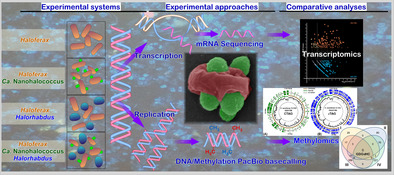

## INTRODUCTION

Studies of epigenetic modifications have advanced our understanding of the mechanisms of soft non‐Mendelian coding of phenotypic properties in microorganisms through chemical modifications of genomic DNA. Enzymatic methylation of nucleosides in genomic DNA is the most well‐known type of epigenetic modification, common to all domains of living organisms, from bacteria and archaea to higher eukaryotes, including humans. DNA methylation is catalysed by S‐adenosylmethionine (SAM)‐dependent methyltransferases (MTases), a vast, highly diverse superfamily of enzymes widespread in cellular organisms (Casadesús & Sánchez‐Romero, 2022). These enzymes likely appeared in prokaryotes at the root of the tree of life (Harris & Goldman, 2020). However, while DNA methylation has been extensively studied in bacteria and eukaryotes, its role in archaeal biology remains poorly understood. The best‐studied role of DNA methylation in prokaryotes is in the context of restriction‐modification (RM) systems. Methylation of genomic DNA by MTases at specific motifs prevents genome cleavage by cognate restriction endonucleases (REases) recognizing the same DNA motifs. This defence mechanism against bacteriophages and conjugative plasmids is common in both bacteria (Ershova et al., 2015) and archaea (Aguirre Sourrouille et al., 2022; Fullmer et al., 2019). The RM systems, along with CRISPR‐Cas systems, act respectively as innate and adaptive immune responses in prokaryotes (Dimitriu et al., 2020; Safari et al., 2020). However, the frequent detection of fully functional standalone or solitary MTases in bacterial genomes cannot be simply explained by the retention of inactive parts of former RM systems. For example, m6A GaTC methylation controlled by the solitary DAM methylase family is characteristic of many enterobacteria but has also been found in phylogenetically distant streptococci (Korotetskiy et al., 2023) and even in archaea (Couturier & Lindås, 2018). In prokaryotes, evidence has been accumulating for the involvement of nucleotide methylation in gene expression and cell cycle regulation, DNA repair, control of DNA‐protein interactions, and phenotypic adaptation through stationary phasing of bacterial populations (Reisenauer et al., 1999; Casadesús & Lowe, 2006; Vasu & Nagaraja, 2013; Mohapatra et al., 2014; Huss et al., 2016; Sánchez‐Romero & Casadesús, 2020). Frequently, bacterial genomes are methylated at several canonical DNA motifs by various MTases that attach methyl groups to the amino groups of the 6th carbon atom of adenine (6 mA methylation), the 4th amino group of cytosine (4mC), or, less commonly in prokaryotes but typical of eukaryotes, to the 5th carbon of cytosine (5mC) (Seong et al., 2022). Closely related microorganisms often differ in methylation patterns due to the frequent replacement of RM systems by horizontal gene transfer (Harris & Goldman, 2020; Korotetskiy et al., 2023). Therefore, methylation patterns can serve as markers for tracing individual strains in complex microbial communities or assembling genomes from metagenomic DNA reads (Korotetskiy et al., 2023; Seong et al., 2022; Wilbanks et al., 2022).

Although genes encoding REases have been found in the genomes of many archaea (Fullmer et al., 2019; Ouellette et al., 2018), the expected endonuclease activity has been experimentally proven for only a handful of them (Zatopek et al., 2021). However, it is possible that the active RM systems found in archaea are not autochthonous for these organisms. Thus, it was shown that the plasmid‐encoded type II RM system MthTI, present in the *Methanobacterium thermoformicicum* THF genome, was horizontally acquired from *Neisseria* bacteria (Nölling & de Vos, 1992). Notably, MTases are generally more abundant in archaeal genomes compared to REases. Therefore, it has been suggested that they may additionally play other biological roles in archaea (Fullmer et al., 2019; Harris & Goldman, 2020; Ouellette et al., 2018).

Modern SMRT PacBio and Oxford Nanopore sequencing technologies have simplified methylomics studies by calling nucleotide modifications in parallel with base calling (Nordlund, 2020). Analysis of the SMRT reads perfectly reproduces the methylation patterns of archaeal genomes predicted by traditional methylomics methods such as droplet‐based bisulfite sequencing, cleavage assays targeting methylated sites, and dot‐blot immunoassays with monoclonal primary antibodies raised against m6A methylation (Couturier & Lindås, 2018; Flusberg et al., 2010).

This study aimed to analyse methylation patterns of *Haloferax lucertense* SVX82, the complete genome sequence of which was recently published (La Cono et al., 2023). There were several reasons for choosing this archaeon for the current study. First, representatives of this genus are widely recognized as model organisms, including for the study of archaeal methylomics. Different RM systems have been reported in different *Haloferax* species (Aguirre Sourrouille et al., 2022; DasSarma et al., 2019; Harris & Goldman, 2020; Marinov et al., 2023; Ouellette et al., 2018; Ouellette et al., 2020; Pérez‐Arnaiz et al., 2020). The most common in *Haloferax* is the Mrr‐RM system, found in the genomes of multiple *Haloferax* species. This MTase adds methyl groups to cytosine residues at CTAG motifs. Other reported MTases are strain‐specific. It has been suggested that m4C CTAG methylation in *Haloferax* contributes to several housekeeping functions, including gene regulation, replication initiation, and DNA repair, resembling the role of m6A GATC methylation in enterobacteria (Ouellette et al., 2018). The avoidance of CTAG palindromes in the genomes of many haloarchaeal viruses, which attack *Haloferax* and other haloarchaea, indirectly suggests that Mrr‐RM systems are actively involved in antiviral defence (Aguirre Sourrouille et al., 2022; Dyall‐Smith et al., 2020; Liu et al., 2021). However, it is worth noting that the endonuclease activity of the cognate Mrr REase at CTAG sites has not yet been experimentally demonstrated in archaea.

Another reason for selecting *H. lucertense* for this study is that this extremely halophilic archaeon can be obtained from salt lakes and seawater evaporation ponds as part of stable three‐membered, xylan‐degrading consortia (La Cono et al., 2023; Reva et al., 2023). Notably, *H. lucertense* cannot degrade xylan on its own, and, to survive on a xylan diet alone, this archaeon must scavenge oligosaccharides produced by another member of the consortium, namely, the xylan‐hydrolyzing *Halorhabdus* sp. In the described haloarchaeal consortia, the *H. lucertense* cells host ectosymbiotic DPANN nanohaloarchaea, that is, *Ca* *ndidatus* Nanohalococcus occultus SVXNc, which acts as an active ecophysiological component, albeit *by proxy*, of microbial communities, utilizing xylan (the third most abundant biopolymer on Earth, after cellulose and chitin) in hypersaline environments (La Cono et al., 2023; Reva et al., 2023). The symbiotic, parasitic, or predatory lifestyle of phylogenetically versatile ultra‐small bacteria and archaea is supported by the producing giant surface proteins like the 9409 aa SPEARE protein (SVXNc_0300) encoded in the *Ca*. N. occultus SVXNc genome (Reva et al., 2023). These giant proteins likely create large pores connecting the cytoplasm of the host cell and the symbiont (Hamm et al., 2023; West‐Roberts et al., 2023), allowing the exchange of protein effectors including methyltransferases. This hypothesis will be tested in this study. Studying changes in methylation patterns of *H. lucertense* genomic DNA, collected from different consortia and under different growth conditions, may shed light on the biological role of DNA methylation in archaea.

Like many other *Haloferax* species, the *H. lucertense* SVX82 strain contains three plasmids, which made it possible to synchronously compare patterns of chromosome and plasmid methylation in this archaeon. The functional significance of DNA methylation can be gleaned from the distribution of methylated sites in both coding and non‐coding regions, as well as in regions proximal to the transcription start sites (TSSs) (Couturier & Lindås, 2018; Marinov et al., 2023). In this study, we used several statistical approaches to analyse the distribution of methylated sites and unmethylated canonical motifs in the protein‐coding regions, in the 75 bp‐long TSS upstream regions, and non‐coding sequences of the *H. lucertense* SVX82 genome. Additionally, gene expression data were also superimposed on the methylation patterns. The study aimed to compare the distribution of methylated motifs controlled by allochthonous and endogenous MTases and to assess their possible involvement in gene regulation. Another objective was to analyse how the ectosymbiotic and xylanolytic members of the consortium affect the methylation patterns in the *H. lucertense* SVX82 genome under different growth conditions.

## EXPERIMENTAL PROCEDURES

### Cultivation conditions

For the axenic culture of *H. lucertense* SVX82 (experiment I), d‐xylose‐using (*H. lucertense* SVX82 + *Ca*. Nanohalococcus occultus SVXNc, experiment II), xylanolytic (*Halorhabdus* sp. SVX81 + *H. lucertense* SVX82, experiment III) and for the xylanolytic three‐member consortium (*Halorhabdus* sp. SVX81 + *H. lucertense* SVX82 + *Ca*. Nanohalococcus occultus SVXNc, experiment IV), the previously described LC liquid mineral medium (La Cono et al., 2020) was used. After sterilization (121°C, 20 min) and cooling, the pH was adjusted to 7.2 by the addition of sterile 1 M KOH. The medium was further supplemented with 1 mL L^−1^ acidic and 1 mL L^−1^ alkaline W/Se trace metal solutions, 1 mL L^−1^ vitamin mix (Pfennig & Lippert, 1966), and 50 mg L^−1^ yeast extract. As a substrate for growing both pure culture and consortia, containing *Halorhabdus* sp. SVX81 sterilized xylan from beechwood (Megazyme, catalogue number P‐XYLNBE‐10G) was added at a final concentration of 1.5 g L^−1^. Since xylan cannot serve as a source of carbon and energy for *H. lucertense* SVX82, 10 mM d‐xylose was added to its axenic and symbiont‐containing binary cultures. As we mentioned earlier, simple aerobic plating on Petri dishes of the lowest positive dilution of xylanolytic enrichment effectively eliminated nanohaloarchaea and resulted in axenic cultures of both *Halorhabdus* sp. SVX81 and *H. lucertense* SVX82 (La Cono et al., 2023). All four cultures (axenic *H. lucertense* SVX82, two binary, and one trinary) were incubated in 120 mL serum vials (in triplicate) statically (to maintain microaerobic conditions) at 40°C, and their growth was observed over a 240 h period. Following a previously described approach (La Cono et al., 2023), halo‐ and nanohaloarchaea were quantified using two different assessment methods. For both haloarchaea, a classical serial dilution/plating approach was applied to monitor their cell densities. The latter approach was used to avoid possible inconsistencies of the DNA‐based qPCR method in cell count results that could be associated with the well‐known polyploidy found in many *Haloferax* species (Lange et al., 2011). In turn, the previously described qPCR method for calculating *Ca*. Nanohalobium cells (La Cono et al., 2020) were used with minor modifications to determine the relative density of *Ca*. Nanohalococcus cells. Briefly, DNA for qPCR was extracted from 2.0 mL of co‐cultures containing nanohaloarchaea, and after quality control using both a NanoDrop ND‐1000 spectrophotometer (Euroclone, Milan, Italy) and a Qubit 3.0 fluorometer (Thermo Fisher Scientific, Milan, Italy) and electrophoresis in a 1.0% (w/v) agarose gel. qPCR was performed with SYBR Green on an ABI Prism 7300 Real‐time PCR System (Applied Biosystems, Foster City, CA, USA). All amplifications were checked for specificity with dsDNA melt curves and samples exhibiting multiple products were not considered in the analysis. The SVXNc 16S rRNA gene‐specific primers (Nhc_1018F 5′‐TGTGAAGTGTCCGGTTAAGT‐3′ and Nhc_1134R 5′‐GCTCCTTCCTCTGTCTTATC‐3′) were designed using Primer Express software, version 2.0 (Applied Biosystems, Foster City, CA, USA). To obtain DNA standards for precise quantification of cell densities, a single clone (colony) containing the 16S rRNA gene of *Ca*. Nanohalococcus occultus SVXNc in pGEM‐T Easy Vector (Promega, Madison, WI, USA) was grown overnight at 37°C and plasmid was subsequently purified using the NucleoBond Xtra Midi KIT (Macherey‐Nagel). Serial dilutions (up to 10^9^ copies μL^−1^) of the plasmid were prepared and used for the qPCR in triplicate to generate a standard curve for sample quantification as previously described (La Cono et al., 2020). Each 25 μL reaction contained 50 ng of DNA isolated from grown cultures, 12.5 μL of SYBR Green Master Mix (ThermoFisher), and 200 nM of each primer. The qPCR protocol included the following steps: an initial denaturation step at 95°C for 10 min, followed by 45 cycles of denaturation at 95°C for 15 s, and annealing/elongation at 60°C for 60 s. A dissociation step was added to check for primer‐dimer formation. After confirming that the obtained values fell within the optimal range (>96%), the PCR amplification efficiency was calculated from the slope of each curve, as described previously (La Cono et al., 2020). All experiments were performed in several independent repetitions.

### Detection of endonuclease activities

Three cultures corresponding to experiment I (axenic culture of *H. lucertense* SVX82 grown on d‐xylose), experiment II (binary culture of *H. lucertense* SVX82 + *Ca*. N. occultus SVXNc, grown on d‐xylose), and experiment IV (trinary culture of *H. lucertense* SVX82 + *Ca*. N. occultus SVXNc + *Halorabdus* sp. SVX81 grown on xylan) were incubated at 40°C for 240 h as described in ‘Cultivation conditions’ section. A fresh biomass pellet from 2 mL of grown cultures was obtained by centrifugation at 10,000 × g for 15 min at 4°C. After pellet resuspension in 20 mM Tris–HCl (pH 7.5), 1.0 M NaCl, 20 mM MgCl_2_, and 1 mM 2‐mercaptoethanol, three freeze–thaw circles were performed followed by sonication using a Vibracell Bioblock Scientific Sonicator 75,115 (Sonics & Materials). Three bursts of 30 s each were made at a cycle duty of 50%. The resulting cell lysates were clarified by centrifugation at 13,000 × g for 20 min at 4°C, and 1 μg of non‐methylated lambda phage DNA (Sigma‐Aldrich) was added to 20 μL of the supernatant. After incubation at 37°C for 2 h, restriction products were checked by electrophoresis in a 1.0% (w/v) agarose gel.

### Nucleic acids (DNA and RNA) extraction and sequencing

Genomic DNA from both pure culture and three *H. lucertense* SVX82‐containing consortia (Supplementary Table S1) was extracted from 5.0 mL of the grown culture using a GNOME DNA KIT (MP Biomedicals, USA). The extracted DNA was dissolved in 50 μL of TE buffer (10 mM Tris–HCl, 1 mM EDTA [pH 7.5]) and quantified using a NanoDrop ND‐1000 spectrophotometer (Celbio) and a Qubit 3.0 fluorometric quantification system (Thermo Fisher Scientific). Additionally, the size and quality of the extracted DNA were checked by electrophoresis in 1.0% agarose gel. Whole genome shotgun sequencing of the tripartite, two bipartite consortia, and the axenic cultures was done by FISABIO (Valencia, Spain) using the Illumina and SMRT PacBio Sequel II system 2.0 platforms.

As for the extraction of RNA, after centrifugation at 10,000 × *g* for 20 min, biomass was collected from 5 mL of grown cultures and total RNA was extracted using MasterPure Complete DNA and RNA purification KIT (Epicentre). Total RNA was stored in isopropanol at −20°C before precipitation. Total RNA was resuspended in 50 μL of RNase‐free water and treated with a TURBO DNA‐free KIT (Ambion) to eliminate any residual DNA from the final elution. The quality and concentration of RNA samples were determined using the Qubit 3.0 fluorometric quantification system (Thermo Fisher Scientific, Italy). The metatranscriptome analysis was performed by FISABIO (Valencia, Spain) using the Illumina® NextSeq Mid Output platform (San Diego, CA, USA) with 2 × 100 bp short insert paired‐end libraries (NextSeq Reagent Kit v2.5). FISABIO also performed the quality assessment and the sequence joining (forward R1 and reverse R2). Quality assessment was performed with the PRINSEQ‐lite program using the following parameters: min_length: 50 bp; trim_qual_right: 30 bp; trim_qual_type: mean; and trim_qual_window: 20 bp.

### Electron cryotomography data collection and processing

To perform electron cryotomography (cryo‐ET), the consortium containing *H. lucertense* SVX82 and *Ca*. N. occultus SVXNc was fixed with 0.125% glutaraldehyde to preserve the interaction between the host and the *Ca*. N. occultus SVXNc symbiont. Subsequently, cells were washed quickly in PBS. This step was done rapidly to prevent any lysis of cells due to changes in the osmolarity of the medium during washing. The cultures were subsequently mixed with 10 nm colloidal gold beads (Ted Pella, CA, USA) pre‐coated with 1% BSA. About 4 μL of the sample mixture was added to a glow‐discharged copper R2/2 Quantifoil holey carbon grid (Electron Microscopy Sciences, PA, USA). Grids were blotted using a Vitrobot (Mark IV, FEI Thermo Fisher Scientific) and plunge frozen in liquid ethane. Grids were imaged under cryogenic conditions, using a Transmission Electron Microscope (FEI Titan Krios G4, 300 KeV FEG; Thermo Fisher Scientific) equipped with a Gatan K3 Summit direct electron detector. An energy filter (GIF, Gatan) with a slit width of 20 eV was used during the operation, and images were collected in electron counting mode. Tilt series were collected between –60° and +60° at 2° increments using the FEI Tomography 5 data collection software. The cumulative total dosage was 100 e^−1^ Å^−2^, with a defocus of −8 μm and a pixel size of 3.4 Å.

Tilt series were aligned with IMOD. Aligned (and binned to 2 k) tilt series were then used for SIRT reconstruction by Tomo3D (Agulleiro & Fernandez, 2015). Tomograms were segmented using a U‐Net convolutional neural network‐based software (Ronneberger et al., 2015). Tomograms were loaded into Dragonfly and filtered using the histogram equalization filter and a 3D Gaussian filter to enhance the contrast. Multi‐ROI training output was generated by hand segmenting the feature of interest in a desired box size. This trained dataset was subsequently used for unsupervised and unbiased segmentation.

### Identification of methylated sites and canonical methylation motifs in sequenced genomes

The long DNA reads generated by SMRT PacBio Sequel II system 2.0 were aligned using the program *pbmm2* of the package smrtlink_10.1.0.119588 against the genome sequences of the archaeal strains available at NCBI under the following accession numbers: *Halorhabdus* sp. SVX81 – CP104322; *H. lucertense* SVX82 – CP104741‐44; and *Ca*. Na. occultus SVXNc – CP104395. Calling the methylated sites and identification of canonical motifs were performed using the programs *ipdSummary* and *motifMaker* of the package smrtlink_10.1.0.119588. The program was run on a computer cluster with 96 cores, 3 TB of RAM, and qsub 6.1.2. The *qsub* job submitting script is shown in the listing below:

*pbmm2 index reference_seq.fasta reference_seq.fasta.mmi*

*pbmm2 align ‐‐sort reference_seq.fasta.mmi source_PacBio_reads.xml aligned.bam*

*ipdSummary aligned.bam ‐‐reference reference_seq.fasta ‐‐identify m6A,m4C ‐‐gff dnamod.gff*

*motifMaker find ‐‐fasta reference_seq.fasta ‐‐gff dnamod.gff ‐‐minScore 20 ‐‐output canonical.motifs.csv*

Methylation recognition sites predicted by the *motifMaker* program were termed ‘canonical motifs’ in this article. In‐house Python scripts were developed to visualize and analyse the distribution of methylated and non‐methylated canonical motifs. It must be noted that the reliability of calling epigenetically modified nucleotides significantly depends on sequencing depth, which must be at least 30 DNA reads aligned against a nucleotide position. The rate of false‐negative predictions of methylated sites increases when sequencing depth is low. The depths of aligned PacBio reads per genome are shown in Supplementary Table S1. Per base sequencing depths in alignments of PacBio reads were controlled by the *depth* function of Samtools‐1.18 (https://github.com/samtools/samtools/releases/). It was found that sequencing depth dropped below 30 in several genomic regions, especially in the plasmids. To avoid any biases due to uneven sequencing depth, all the genomic regions showing sequencing depth below 30 in at least one of the four experiments were masked from the analysis. In total, 789,186 bp were masked in the genome of *H. lucertense* SVX82, comprising 21%, including 409,186 bp of the chromosomal sequence (15%) and 380,080 bp of the plasmid sequences (38%).

### Differential gene expression analysis

Transcriptional analysis was performed using the Bioconductor software package version 3.17 (http://www.bioconductor.org/), which was run on an R‐3.4.4 installation. The generated RNA sequences were aligned against sequences of the reference genomes indexed by Rsubreads. Aligned reads were sorted with Samtools‐1.18. The annotation of the reference genomes was provided in GFF format. Reads overlapping predicted coding sequences (CDS) were counted by the featureCounts function of the Bioconductor Rsubread package. The DESeq2 and GenomicFeatures programs of the Bioconductor package were used to normalize the counts by the total numbers of reads in the samples and by lengths of CDS and then compared to produce statistical values of gene expression (baseMeans), expression fold change values in comparison between different datasets, and *p*‐values for detected differences. To compare gene expression levels across different experiments, RPKM (reads per kilobase per million mapped reads) values of gene expression were calculated:

$$\text{RPKM}=\begin{array}{l} \text{Number}\_\text{of}\_\text{reads}\_\text{mapped}\_\mathrm{per}\_\text{gene}\\ \times 10^{9}/\text{total}\_\text{mapped}\_\text{reads}/\text{gene}\_\text{length} \end{array}$$

### Statistical analysis and validation

MTase genes in the sequenced genomes of *H. lucertense* SVX82, *Halorhabdus* sp. SVX81, and *Ca*. N. occultus SVXNc was identified using the REBASE database (https://ngdc.cncb.ac.cn/databasecommons/database/id/42) (Roberts et al., 2015). A protein sequence similarity search through the NCBI *nr* database was performed using the BLASTP web‐based tool (https://blast.ncbi.nlm.nih.gov/Blast.cgi). Horizontally transferred genetic islands in the genome of *Halorhabdus* sp. SVX81 was identified using SeqWord Genomic Island Sniffer (Bezuidt et al., 2009).

For a statistical assessment of the randomness of the distribution of methylated and unmethylated canonical motifs on the chromosome, four‐cell contingency table statistics were applied (Fleiss et al., 2013). Contingency tables were designed to test whether the presence of the ectosymbiont in consortia II and IV (Table S1) affects the host's methylation patterns, compared to those obtained in *H. lucertense* SVX82 grown axenically, or in the binary culture with *Halorhabdus* sp. SVX81 (consortia I and III, respectively). Numbers of methylated or unmethylated canonical motifs in *H. lucertense* SVX82 genome co‐occurring in two or three different consortia, were used to generate contingency tables, as explained in Table 1. Canonical motifs that were methylated or not methylated in all the experiments, or those that occurred only in one experiment, were not considered in the contingency tables.

**TABLE 1:** Formulation of the contingency tables for statistical evaluation of dependences of nucleotide methylation patterns of *Haloferax lucertense* SVX82 grown at different experimental conditions.

(A) Contingency table to evaluate the influence of cultivation of *H. Lucertense* SVX82 on xylan with *Halorabdus* sp. SVX81 (consortia III and IV) on the methylation patterns		
	I	III
II	^a^ *D*_1_ = *N*_{I:II}_ + ½ *N*_{I:II:III}_ + ½ *N*_{I:II:IV}_	*D*_2_ = *N*_{II:III}_ + ½ *N*_{I:II:III}_ + ½ *N*_{II:III:IV}_
IV	*D*_3_ = *N*_{I:IV}_ + ½ *N*_{I:II:IV}_ + ½ *N*_{I:III:IV}_	*D*_4_ = *N*_{III:IV}_ + ½ *N*_{I:III:IV}_ + ½ *N*_{II:III:IV}_

(B) Contingency table to evaluate the influence of cultivation of *H. lucertense* SVX82 with the ectosymbiont Ca. N. occultus SVXNc (consortia II and IV) on the methylation patterns		
	IV	I
II	*D*_1_ = *N*_{II:IV}_ + ½ *N*_{I:II:IV}_ + ½ *N*_{II:III:IV}_	*D*_2_ = *N*_{I:II}_ + ½ *N*_{I:II:III}_ + ½ *N*_{I:II:IV}_
III	*D*_3_ = *N*_{III:IV}_ + ½ *N*_{I:III:IV}_ + ½ *N*_{II:III:IV}_	*D*_4_ = *N*_{I:III}_ + ½ *N*_{I:II:III}_ + ½ *N*_{I:III:IV}_

^a^ *D*_1,2,3,4_ – respective four contingency table numbers; *N*_{i}_ – numbers of methylated or not methylated sites on the chromosome or the plasmids of *H. lucertense* SVX82 shared between different experiments (*i*‐parameter). For example, *N*_{I:II}_ – number of genomic sites methylated in the same way in consortia I and II. The description of the composition of the different consortia is given in Appendix Table S1.

The non‐randomness of the distribution of patterns of DNA methylation was verified using contingency table *p*‐value statistics, implemented in the *stats.chi2_contingency* function of the SciPy 1.11.1 library for Python 3.11.4. The *chi2_contingency* function calculates chi^2^ and *p*‐statistics for the provided contingency tables, as illustrated in Table 1, and returns expectation frequency tables under the assumption of a random distribution of methylated sites. The linkage disequilibrium (LD) parameter was estimated as the difference between the observed and expected frequencies in the top‐left cells of the contingency tables (Table 1). LD was normalized by the minimum expected number in the top‐left and bottom‐right cells when LD is negative, or the minimum number in the top‐right and bottom‐left cells when LD is positive. Normalized LD values vary from −1.0 to +1.0. The absolute value of LD indicates the level of bias between methylated sites under two different conditions and is deemed statistically reliable if the respective *p*‐values are ≤0.05. The sign of LD shows whether the nucleotide sites are co‐methylated (positive LD) or methylated (negative LD) under the two conditions.

The binomial test, implemented in the SciPy 1.11.1 library for Python 3.11.4, was used to estimate the *p*‐values of deviations between the observed and expected frequencies of methylated and unmethylated canonical motifs in different functional regions of the genome. The Pearson rank correlation between the numbers of methylated sites in gene bodies and gene expression values was calculated using the *scipy.stats.spearmanr* function (SciPy 1.11.1). Expected frequencies were estimated based on the assumption of an unbiased random distribution proportional to the lengths of sequences in different functional categories. For this analysis, CDS locations were obtained from the annotated whole genome sequence of *H. lucertense* SVX82 published on NCBI (BioSample PRJNA865582). The following functional regions were considered: the fraction of coding sequences *f*_cds_ = Σ(CDS_lengths)/(2 × genome length); the fraction of 75 bp TSS‐upstream regions *f*_prom_ = 75 × Σ(CDS)/(2 × genome length), and the fraction of non‐coding sequences *f*_nс_ = 1 – *f*_cds_ – *f*_prom_. It should be noted that methylation of nucleotides occurs on one of the two DNA strands, which is why the total length of the genome is twice the length of one DNA strand. Non‐coding sequences include the reverse‐complement strands of the CDS sequences. Start and stop codons were considered as part of the coding sequences.

## RESULTS

### Genome methylation patterns

Three stable consortia of the selected archaea, along with the axenic culture of *H. lucertense* SVX82, were grown under appropriate conditions (see Appendix Table S1). Their nucleic acids were extracted, and DNA was sequenced using the SMRT PacBio Sequel system, while directional RNA‐seq cDNA libraries were sequenced on the Illumina HiSeq platform. The generated DNA and cDNA reads (referenced in Appendix Table S2) were used respectively for whole genome assembly, methylomics, and transcriptomics.

The complete genome sequence and reconstructed methylation pattern of the ectosymbiotic DPANN archaeon, *Ca*. N. occultus SVXNc, reported in a previous publication (Reva et al., 2023), was confirmed in this study. This genome is methylated at cytosine residues on both DNA strands in 138 out of the 140 pseudo‐palindromic GDG*c*HC motifs found in the genome. Hereinafter, the methylated residues in the canonical motifs are denoted in lowercase italics. One GTGCAC locus and its reverse complement locus located within the *ppsA* coding sequence, remained unmethylated in all replicates of the experiments. Genome annotation and the REBASE database search predicted two Dcm‐like type II DNA methyltransferases (MTases), SVXNc_0487 and SVXNc_0752, which could potentially form a restriction‐modification (RM) system together with a single Mrr‐type REase (SVXNc_0757). These genes showed a significant level of expression in all experiments; however, only one canonical motif, GDG*c*HC, was identified.

Multiple methylated adenine and cytosine residues have been found in the xylanolytic *Halorabdus* sp. SVX81. The REBASE database predicted one type I RM system, composed of the REase subunit (SVXHr_1196), the DNA motif recognition subunit HsdS (SVXHr_1198), and MTase HsdM (SVXHr_1199); one type II RM system, composed of the MTase SVXHr_0457 and REase SVXHr_0458; and one solitary type II MTase SVXHr_0429. Although all these genes showed a significant level of expression and strong signals of methylated nucleotides were detected in the SMRT reads, no conserved canonical motifs supporting this methylation were identified.

A complex pattern of methylated adenine and cytosine residues has been identified in *H. lucertense* SVX82. Methylation occurred at several canonical motifs, namely, *c*TAG and C*c*TTG cytosine methylation, and two motifs associated with adenine methylation: GTCG*a*GG and GRAGA*a*G. The numbers of methylated and unmethylated canonical motifs found in the chromosome and the three plasmids of *H. lucertense* SVX82 are shown in Table 2.

**TABLE 2:** Numbers and fractions of methylated motifs in the genome of *Haloferax lucertense* SVX82.

Motif	Number of motif loci per genome^a^	Number of methylated sites			
		I Xylose (Hfx)	II Xylose (Hfx + Nhc)	III Xylan (Hfx + Hrb)	IV Xylan (Hfx + Nhc + Hrb)
*c*TAG	1554–1556^b^	908 (58.4%)	1024 (65.8%)	908 (58.4%)	1070 (68.6%)
C*c*TTG	2696–2697	2100 (77.9%)	2025 (75.1%)	2039 (75.6%)	2134 (79.2%)
GTCG*a*GG	4747–4748	4238 (89.3%)	4086 (86.1%)	4216 (88.8%)	4259 (89.7%)
GRAGA*a*G	1125–1127	976 (86.7%)	987 (87.6%)	965 (85.7%)	976 (86.7%)
GDG*c*HC	13,569–13,577	57 (0.4%)	449 (3.3%)	144 (1.1%)	330 (2.4%)

^a^ These numbers do not include motifs in the masked regions of the reference genome.

^b^ Number of available canonical motifs may vary from experiment to experiment as the program *ipdSummary* searches for methylated sites in consensus sequences constructed de novo in every program run by mapping the PacBio reads against the reference sequence.

Genome annotation of *H. lucertense* SVX82 and a search in the REBASE database identified one solitary MTase, SVXHx_0753, located on the chromosome. According to the REBASE prediction, this MTase can perform cytosine‐specific methylation of DNA at *c*TAG motifs. The translated protein sequence of SVXHx_0753 showed 82% identity to the *c*TAG‐specific MTase of *Haloferax mediterranei* ATCC 33500 (DasSarma et al., 2019). Two other type II chromosomal MTases, SVXHx_1615 and SVXHx_2221, may also be involved in DNA methylation. Both genes are located within two predicted genomic islands, surrounded by transposases and phage‐like integrases, indicative of horizontal acquisition of these genes (Supplementary Figure S1). SVXHx_2221 is located next to a cognate REase, SVXHx_2220, forming a type II RM system. MTase SVXHx_1615 is part of the MmeI‐like Type III RM enzymes, comprising the N‐terminal REase, central MTase, and C‐terminal target recognition domains. Another putative RM gene cluster, consisting of adenine‐specific MTase SVXHx_3135 and a possible REase SVXHx_3134 was located on the plasmid pSVX82‐1. An additional solitary MTase, SVXHx_5097, was located on the plasmid pSVX82‐3. Plasmid‐born MTases were found within transposable elements (Supplementary Figure S1). Expression of all these genes was confirmed by the total RNA sequencing, except for REase SIVHx_3134, which was transcriptionally silent in all experiments. The chromosomal and plasmid‐born MTases can explain the observed adenine and cytosine methylation at *c*TAG, C*c*TTG, GTCG*a*GG, and GRAGA*a*G motifs predicted by motifMaker.

**FIGURE 1:**
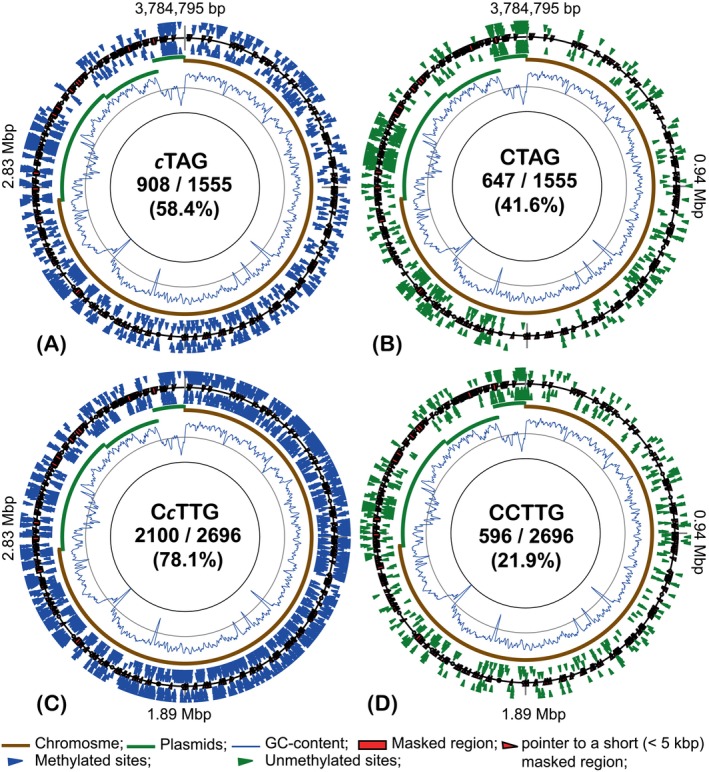
Distribution of methylated *c*TAG (A); unmethylated CTAG (B); methylated C*c*TTG (C); and unmethylated CCTTG (D) motifs in the genome of *Haloferax lucertense* SVX82 grown on 10 mM d‐xylose as an axenic (pure) culture. Methylated and unmethylated loci are indicated by blue and green triangle labels, respectively. External and internal markings indicate respectively the methylation on the direct and the reverse complement DNA strands. Methylated residue in the motif label is depicted in lowercase in italics. The histogram curve shows fluctuations in GC content over a 5000 bp sliding window. Chromosomal and plasmid replicons are shown by solid brown and green arcs, respectively. Genetic regions masked from the analysis due to the unstable depth of sequencing are indicated. The numbers separated by a slash indicate respectively the numbers of unmethylated or methylated loci and the total number of the motifs found in the genome. Below the numbers is the percentage of methylated sites throughout the genome.

A remarkable finding was that only fractions of the available canonical motifs were methylated in the *H. lucertense* SVX82 genome. The distribution of unmethylated CTAG and CCTTG motifs demonstrated a non‐random pattern (Figure 1B, D). Unmethylated canonical motifs were clustered in plasmids and chromosomal regions characterized by a significantly lower GC content than the average for the chromosome (67%). This observation suggests that the binding of *H. lucertense* SVX82 MTases to recognized canonical motifs is modulated by the GC content of the surrounding genomic regions. These regions may have an alternative chromatin conformation, precluding access to MTases. Regardless, this reduction in methylation was not absolute, as many cTAG and CcTTG motifs were methylated in these low‐GC regions (Figure 1A, C).

### Endonuclease activity of the archaeal strains and the consortia

**FIGURE 2:**
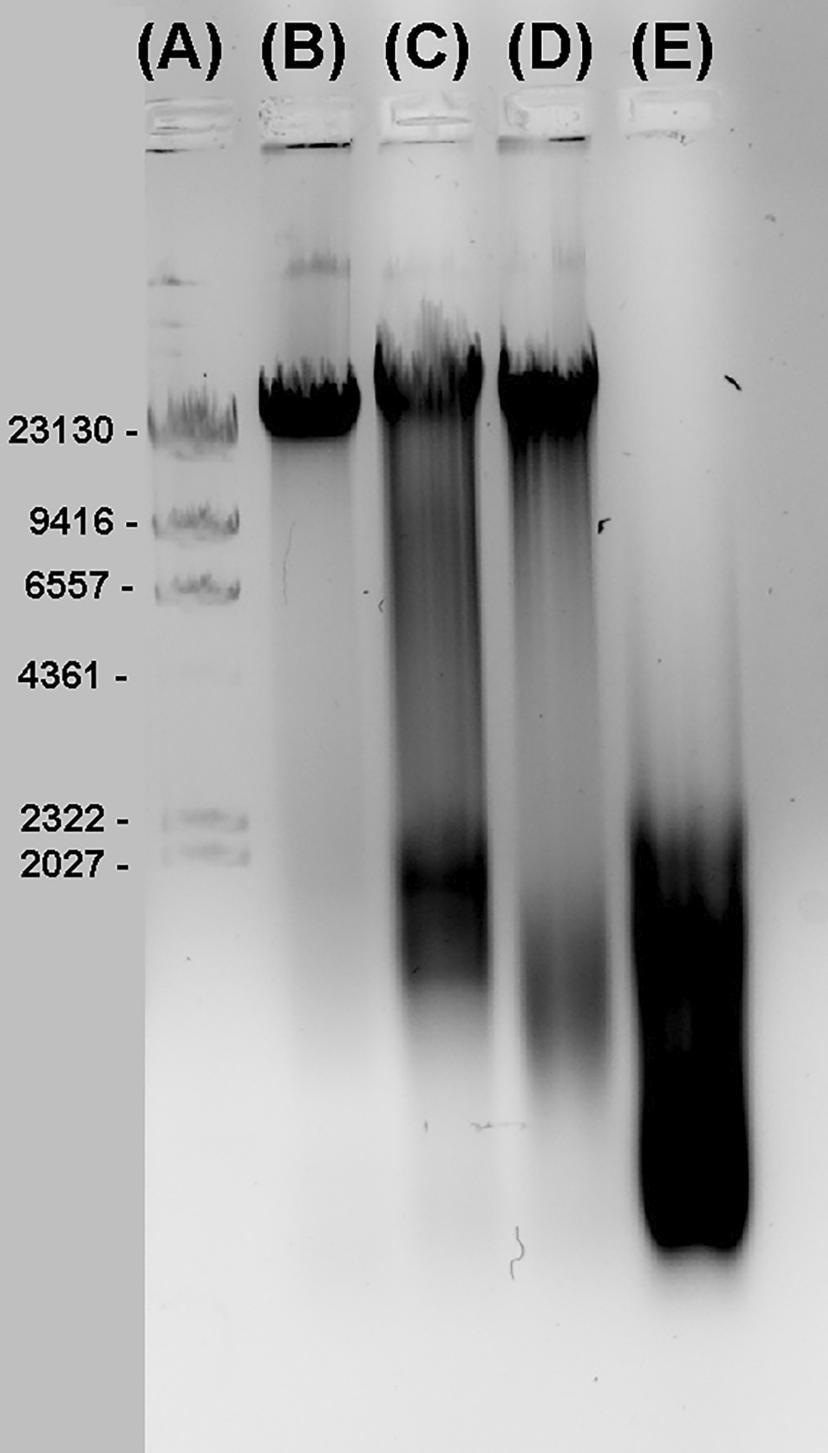
Restriction activity of cell lysates obtained from various archaeal consortia. Lambda DNA *Hind*III ladder (A); Intact unmethylated lambda phage DNA (λDNA) (B); λDNA treated with the cell lysate of consortium I (C), consortium II (D), and consortium IV (E).

The endonuclease activity of the archaeal strains and different consortia was determined experimentally by the cleavage of unmethylated lambda phage DNA (Figure 2). A cell lysate of the *H. lucertense* SVX82 axenic culture moderately cleaved the lambda phage DNA, producing a smear of partially digested fragments with the highest density corresponding to oligonucleotides of 1500–2000 bp. However, a significant amount of the loaded DNA remained intact. DNA cleavage by the cell lysate from the binary culture of *H. lucertense* SVX82 and its symbiont *Ca*. N. occultus SVXNc was more efficient, resulting in shorter fragments of digested DNA (1000—1500 bp long). This result indicates a contribution of the *Ca*. N. occultus SVXNc REase(s) to the total endonuclease activity. Finally, complete digestion of the lambda phage DNA was achieved using the cell lysate from the tripartite consortium comprising *H. lucertense* SVX82, *Ca*. N. occultus SVXN, and *Halorhabdus* sp. SVX81. This experiment demonstrated that all three archaeal strains synthesize active endonucleases.

### Influence of the growth conditions and the archaeal consortia composition on the methylation patterns

Given the observed diversity of canonical methylation motifs in the *H. lucertense* SVX82 genome and the only partial methylation of the available motifs, this organism was deemed to be an optimal model for studying the factors that influence patterns of genome methylation.

#### Cytosine methylation

Cytosine methylation occurred at two canonical motifs: *c*TAG palindromes and non‐palindromic C*c*TTG, with methylation occurring only on one of the DNA strands (hemimethylation). The frequency of unmethylated CTAG sites was higher on the SVX82 plasmids, characterized by an alternative GC composition (Figure 1C and Supplementary Figure S2 A). This suggests that the efficiency of *c*TAG methylation was dependent, at least in part, on the DNA composition. Many of these sites remained unmethylated in all four experiments (Supplementary Figure S2 A,B). The genomic DNA of *H. lucertense* SVX82 obtained from consortia II and IV (grown with the *Ca*. N. occultus SVXNc symbiont) was characterized by fewer unmethylated CTAG sites, that is, higher methylation efficiency at these motifs (Table 2). However, the difference in the number of unmethylated sites in different experiments was statistically insignificant (Supplementary Figure S2 A,B). Cytosine methylation at C*c*TTG motifs also depended on the local GC composition (Figure 1C,D). The fraction of methylated sites was 75%–79% under all conditions (Table 2). The pattern of distribution of unmethylated sites was biased depending on whether the strain was cultivated on xylose or in a consortium with *Halorhabdus* sp. SVX81 on xylan (*p*‐value = 0.026; Supplementary Figure S2 C). The presence of the symbiont does not affect m4C methylation patterns.

#### Adenine methylation

**FIGURE 3:**
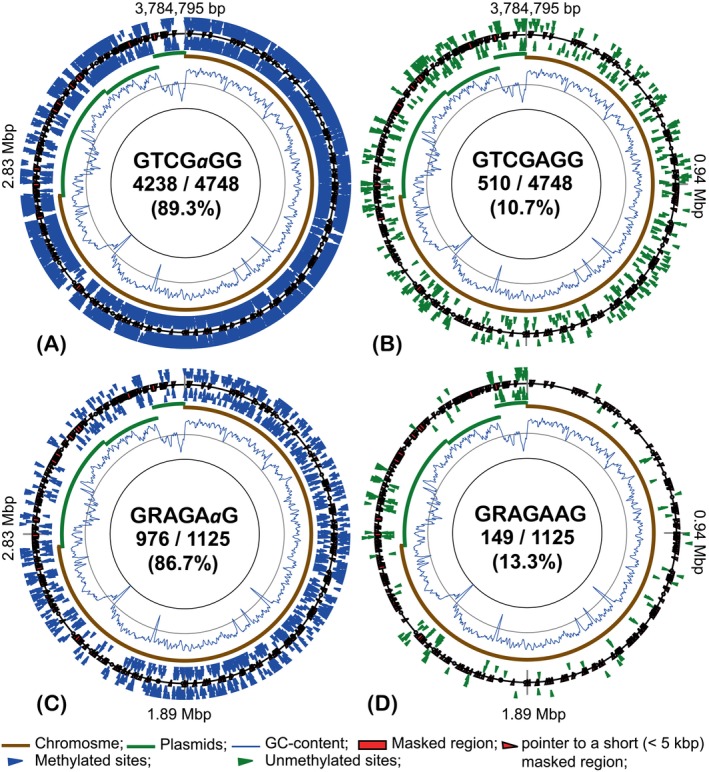
Distribution of methylated GTCGaGG (A); unmethylated GTCGAGG (B); methylated GRAGAaG (C) and unmethylated GRAGAAG (D) motifs in the genome of *Haloferax. lucertense* SVX82 grown on 10 mM d‐xylose as an axenic culture. Methylated and unmethylated loci are indicated by blue and green triangle labels, respectively. External and internal markings indicate respectively the methylation on the direct and the reverse complement DNA strands. Methylated residue in the motif label is depicted in lowercase in italics. The histogram curve shows fluctuations in GC content over a 5000 bp sliding window. Chromosomal and plasmid replicons are shown by solid brown and green arcs. Genetic regions masked from the analysis due to the unstable depth of sequencing are indicated. The numbers separated by a slash indicate respectively the numbers of unmethylated or methylated loci and the total number of the motifs found in the genome. Below the numbers is the percentage of methylated sites throughout the genome.

Adenine methylation in the genome of *H. lucertense* SVX82 occurred at two non‐palindromic canonical motifs: GTCG*a*GG and GRAGA*a*G. The first motif is more abundant in the genome than the second one (Table 2 and Figure 3). As with cytosine methylation, unmethylated adenine residues within the recognized motifs were more frequent in the genomic regions characterized by lower GC content. The fractions of methylation at GTCG*a*GG and GRAGA*a*G were 86%–90% in all four experiments. The distribution of methylated and unmethylated GTCGAGG sites was affected by both: growth on xylan with *Halorabdus* sp. SVX81 (*p*‐value = 0.0) and the presence of symbiotic archaeon *Ca*. N. occultus SVXNc (*p*‐value = 0.024; Supplementary Figure S3). The distribution of methylated and unmethylated GRAGAAG was close to random.

#### Methyltransferase exchange between symbiotic archaea and non‐canonical methylation

**FIGURE 4:**
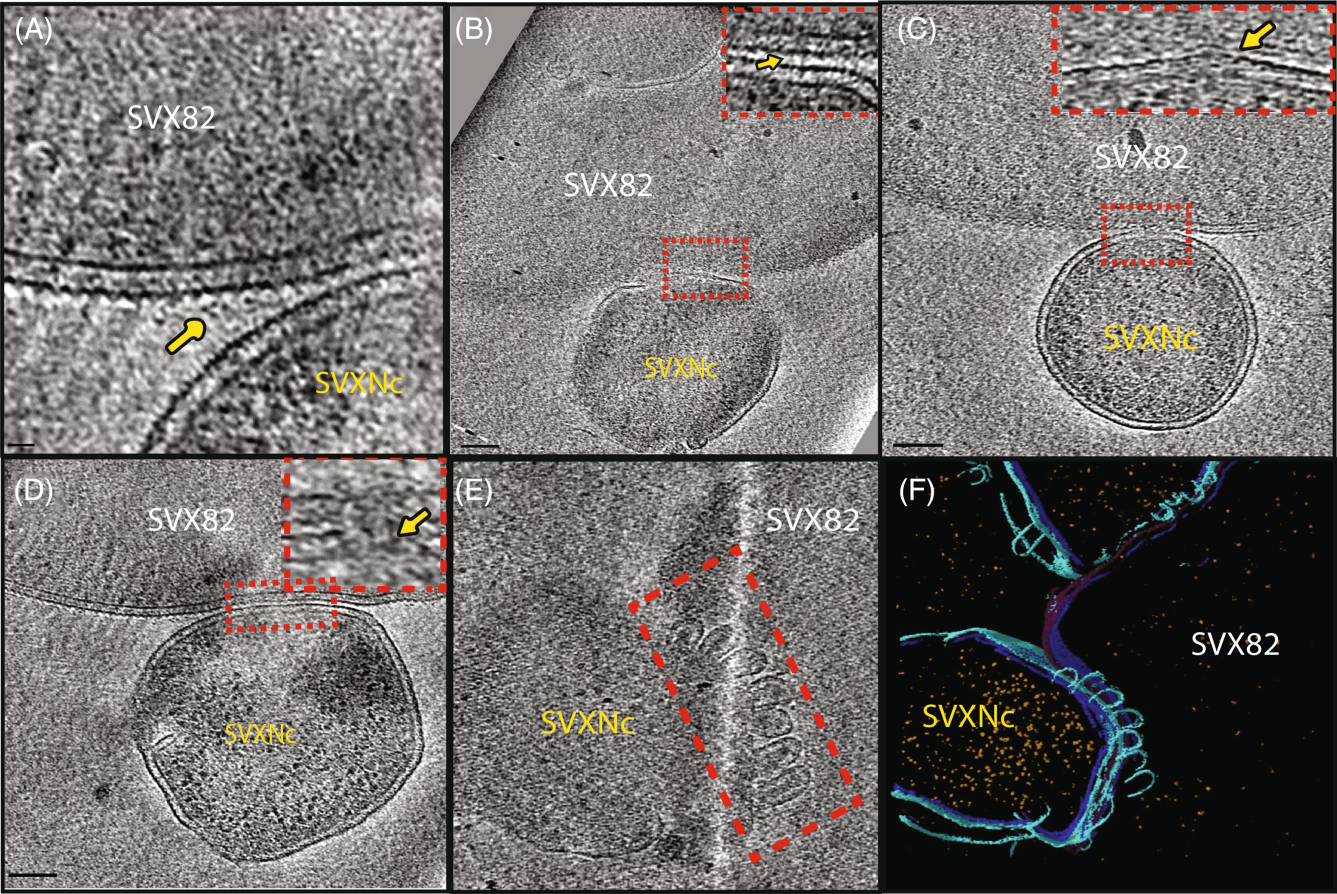
Cryo‐ET analysis of the interaction between the host (SVX82) and DPANNs (SVXNc) in a coculture system. (A) Representative tomographic slice showing proteinaceous densities protruding out of the host cell (SVX82) surface (indicated by a yellow arrow). (B) Tomographic slice showing host‐DPANN interaction. Inset showing host‐DPANN interface bridged by a thin filament‐like density connecting the two cell types. (C) Tomographic slice showing a host‐DPANN interaction. Inset showing an enlarged view of the red‐boxed area. There is a dimple on the host surface and an inverted ‘V’ shaped protrusion on the DPANN surface (D) Representative tomographic slice indicating the enlarged view of the cytoplasmic bridges highlighted in red in the inset. The membrane boundaries of both cells look fuzzy where the cytoplasmic bridge is formed. (E) Tomographic slice showing extensive membrane blebbing from the DPANN SVXNc at the DPANN‐host interface. Blebs are highlighted in the red box. (F) 3D Segmentation analysis highlights key components: Host SVX82 S‐layer (dark purple), inner membrane (dark blue), outer membrane (light blue) ribosomes (yellow). Scale bars indicate (B–D) (100 nm).

It was predicted in previous studies that *Ca*. N. occultus SVXNc, the ectosymbiont of *H. lucertense* SVX82, has an elaborate transmembrane transport system that is likely used to tame and manipulate the haloarchaeal host by secreting various regulatory proteins and exchanging certain metabolites (La Cono et al., 2023; Reva et al., 2023). Here, interactions between *H. lucertense* SVX82 and *Ca*. N. occultus SVXNc cells were studied in their frozen hydrated state using cryo‐ET (Figure 4). In the obtained cryotomograms, the host cell surface displayed proteinaceous ‘antennae‐like’ densities (Figure 4A). These densities could be putative adhesins/receptors driving initial contact formation between the host and the symbiont. Additionally, in some of the tomograms, we noticed a thin filament‐like density bridging the two interacting cells suggestive of intercellular interaction (Figure 4B). At the interface between the host and the symbiont, the host often showed a dimple/dip on the envelope, while the symbiont showed an inverted ‘V’‐shaped protrusion, suggesting a tight association between the host and the symbiont (Figure 4C). Intriguingly, at the contact point between the host and the symbiont, we often saw diffused membrane boundaries and formation of cytoplasmic bridges, measuring 9–11 nm in diameter (Figure 4D). This suggests intercellular material transfer could occur through these cytoplasmic bridges. Another interesting feature we observed at the host‐symbiont interface was intense membrane blebbing from the DPANN cell towards the host (Figure 4E,F, Supplementary Video S1). Whether these membrane blebs are associated with cytoplasmic bridge formation or they have additional roles in material exchange is the subject of ongoing research.

**FIGURE 5:**
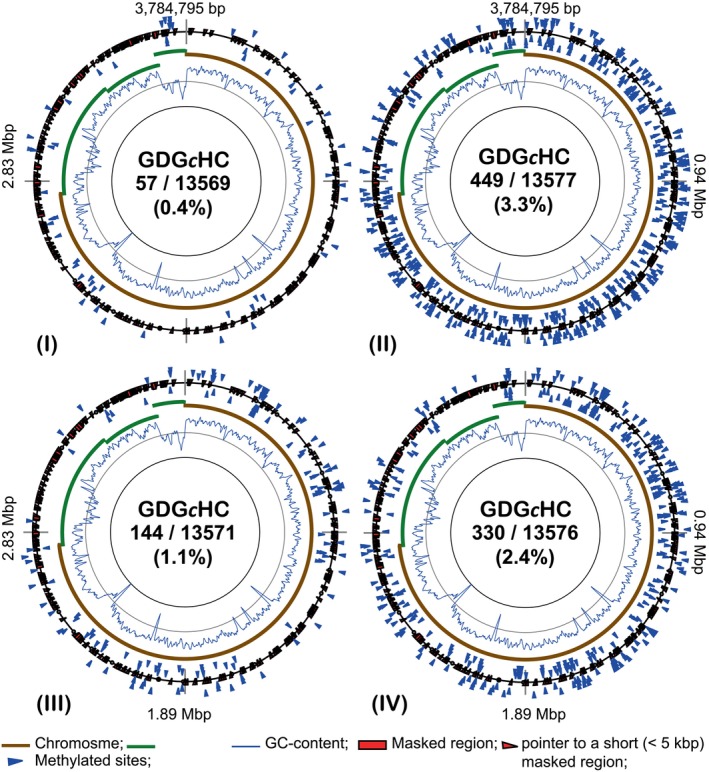
Distribution of GDG*c*HC methylated sites in the *Haloferax lucertense* SVX82 genome when grown in different experiments: (I) pure (axenic) culture on d‐xylose; (II) binary culture with the ectosymbiont *Ca*. N. occultus SVXNc on d‐xylose; (III) binary culture with *Halorabdus* sp. SVX81 on xylan; (IV) trinary culture with *Halorabdus* sp. SVX81 and the ectosymbiont *Ca*. N. occultus SVXNc on xylan. Methylated and unmethylated loci are indicated by blue and green triangle labels, respectively. External and internal markings indicate respectively the methylation on the direct and the reverse complement DNA strands. Methylated residue in the motif label is depicted in lowercase in italics. The histogram curve shows fluctuations in GC content over a 5000 bp sliding window. Chromosomal and plasmid replicons are shown by solid brown and green arcs, respectively. Genetic regions masked from the analysis due to the unstable depth of sequencing are indicated. The numbers separated by a slash indicate respectively the numbers of methylated loci and the total number of GDGCHC motifs found in the *H. lucertense* SVX82 genome. Below the numbers is the percentage of methylated sites throughout the genome.

To test whether the nanohaloarchaeal MTases might be among the proteins transported to the host, the CDG*c*HC methylation (the specific methylation of the *Ca*. N. occultus SVXNc genome) was examined in the *H. lucertense* SVX82 genome. When this haloarchaeon was grown without the symbiont (consortia I and III), only 0.4%–1.1% of the CDGCHC sites present in *H. lucertense* SVX82 were methylated (Figure 5 I, III). The percentage of methylation at the CDG*c*HC sites in the *H. lucertense* SVX82 genome increased to 2.4%–3.3% in consortia II and IV where the symbiont was present (Figure 5 II, IV). This difference was statistically significant for the chromosomal loci (Supplementary Figure S4). The CDG*c*HC methylation patterns were dissimilar to each other. There was not a single CDG*c*HC site methylated in all four experiments, whereas the canonical methylation of the *H. lucertense* SVX82 at the *c*TAG, C*c*TTG, GTCG*a*GG, and GRAGA*a*G was more stable, with multiple sites repeatedly methylated or not methylated under all conditions (compare Supplementary Figure [Link], [Link]).

#### Distribution of methylated and unmethylated motifs among coding, non‐coding, and regulatory genomic sequences

**FIGURE 6:**
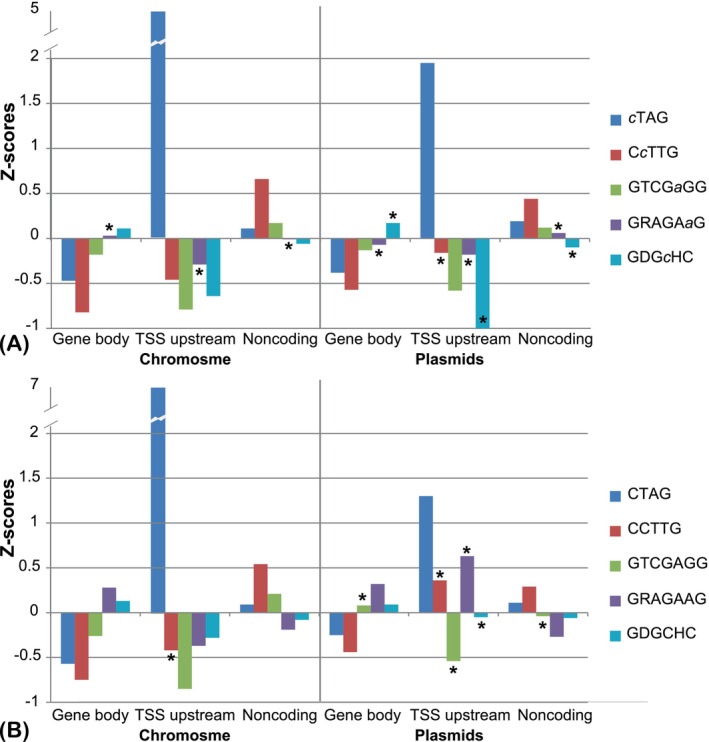
Z‐scores of deviations between observed and expected numbers of methylated (A) and unmethylated (B) motifs in the gene bodies, the 75 bp TSS upstream regions, and in the non‐coding parts of the genome of *Haloferax lucertense* SVX82. Most of the deviations were statistically reliable. Those deviations producing *p*‐values lower than 0.05 are marked by asterisks.

Statistical analysis of the distribution of methylated and unmethylated canonical sites in coding and non‐coding genomic regions may shed light on the possible roles of MTases in gene regulation. The sequence length proportions of coding regions, the 75 bp TSS‐upstream, and non‐coding regions were calculated for the genome of *H. lucertense* SVX82. These proportions were respectively 42%, 3% and 55% for the chromosome, and 40%, 3% and 57% for the plasmids. Randomly distributed motifs should follow these ratios. To check the hypothesis, *z*‐scores of the deviations observed from expected frequencies were calculated using the following equation: *z*‐score = (*F*_obs_ – *F*_exp_)/(*F*_exp_ + 1). The statistical reliability of deviations between the observed and expected numbers was verified by the binomial test. Frequencies of occurrence of methylated and unmethylated canonical motifs in the coding, TSS‐upstream, and non‐coding regions are shown in Figure 6A,B.

**FIGURE 7:**
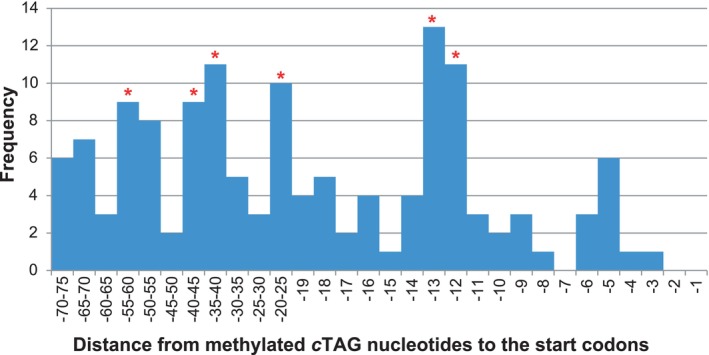
Frequencies of the finding of m4C methylated cytosine residues constituting *c*TAG motifs upstream of the start codons of protein‐coding genes. Statistically reliable frequency increases (*p*‐value ≤0.05) are marked by red asterisks.

The most striking finding was the high frequency of occurrence of methylated and unmethylated CTAG motifs in the 75 bp TSS‐upstream regions of protein‐coding genes, which exceeded the expected number of these motifs by several folds, under the assumption of their random distribution. The distribution of the *c*TAG motifs, methylated in at least one experiment, relatively to the TSS, was uneven (Figure 7). Cytosine residues of the *c*TAG motifs, whether methylated or unmethylated, were higher than expected in the following regions: from −12 to 13 bp, from −20 to 25 bp, from −35 to 45 bp and from −50 to 60 bp. Methylation near the promoter regions may interfere with the binding of transcriptional regulators to these regions. Genes with *c*TAG methylation within 20 bp upstream of TSS are listed in Supplementary Table S3.

All other canonical motifs of *H. lucertense* SVX82 showed a tendency to avoid being located and methylated within the TSS‐upstream regions and in the protein‐coding genes (Figure 6A,B). The C*c*TTG methylation was abundant in non‐coding regions.

#### Distribution of methylated and unmethylated motifs within protein‐coding genes with different levels of expression

Noteworthy results were obtained by comparing the frequencies of methylated and unmethylated canonical motifs in the protein‐coding sequences characterized by different levels of gene expression. Five categories of gene expression were defined. First, transcriptionally silent genes with an average RPKM (reads per kilobase per million mapped reads) value of less than 5 were assigned to the 0‐level expression group. The remaining genes were sorted by their average RPKM values and divided into four quartiles, from low‐level expression genes (quartile 1) to the genes with the highest level of expression (quartile 4). Random distribution expected that the frequency of detection of methylated and unmethylated canonical motifs corresponds to the ratios of the total lengths of genes of the different categories, which were respectively 9.7%, 24.1%, 23.1%, 23.3% and 19.5% for the groups 0–4. *Z*‐scores of deviations of observed frequencies from expected ones were calculated as explained above.

**FIGURE 8:**
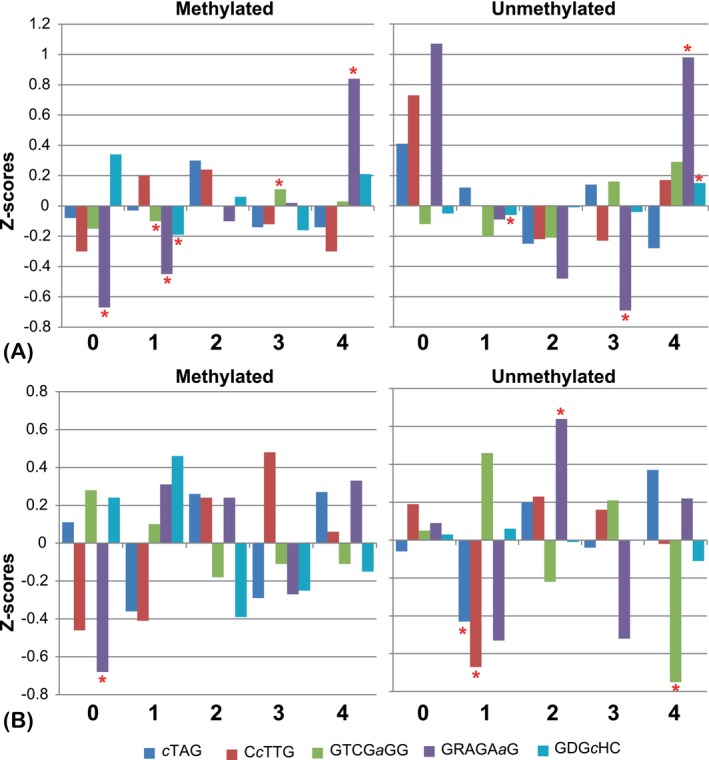
Z‐score values of the observed versus expected frequencies of methylated and unmethylated motifs found in protein‐coding genes of the chromosome (A) and the plasmids (B) of *Haloferax lucertense* SVX82. Genes were grouped into five categories based on gene expression: (1) transcriptionally silent; (2) low expressed; (3) moderately expressed; (4) elevated gene expression; and (5) highly expressed genes. The colours of the bars indicate different methylation motifs. Statistically reliable deviations from expected frequencies (*p*‐value ≤0.05) are marked by red asterisks.

The distribution of methylated and unmethylated motifs found within chromosomal and plasmid protein‐coding regions, across genes with different expression levels, is shown in Figure 8. Unmethylated motifs CTAG, CCTTG and GRAGAAG were abundant in transcriptionally silent genes. This abundance is associated with the lower methylation efficacy of *H. lucertense* SVX82 MTases in the regions with low GC content, as discussed above (see Figures 1 and 3). Silent genes were located in these regions, including virus‐derived genes and pseudogenes that arose from the fragmentation of phage and plasmid insertions.

The adenine methylation pattern at GRAG*a*AG motifs showed clear targeting of highly expressed chromosome‐located genes, but not those located on the plasmids. No correlation with gene regulation was observed for the conditional GRAG*a*AG methylation. The distribution of methylated GDG*c*HC sites, controlled by the symbiont's MTase, was close to random, with a moderate increase in the efficiency of methylation in both transcriptionally silent and the most highly expressed genes. The distribution of GTCG*a*GG methylated motifs was close to random in both coding and non‐coding regions (Figure 6) and across genes with different expression levels (Figure 8).

The methylated GRAGA*a*G motifs tended to be present in highly expressed genes of *H. lucertense* SVX82, with a frequency statistically significantly exceeding expectations. At the same time, the frequency of these motifs in genes exhibiting low expression levels or that were transcriptionally silent was significantly below expectation (Figure 8). A list of genes characterized by high frequencies of GRAGA*a*G motifs is provided in Supplementary Table S4.

## DISCUSSION

In addition to the known function in RM defence systems (Ershova et al., 2015), it is becoming increasingly apparent that DNA methylation by bacterial MTases also plays critical roles in the regulation of gene expression and the genetic phasing of bacterial populations (Casadesús, 2016; Casadesús & Low, 2006; Harris & Goldman, 2020; Huss et al., 2016; Mohapatra et al., 2014; Reisenauer et al., 1999; Sánchez‐Romero & Casadesús, 2020; van der Woude, 2011; Vasu & Nagaraja, 2013). By contrast, the role of DNA methylation in archaea remains poorly understood. Lateral exchange of DNA fragments between bacteria and archaea was reported (Dodsworth et al., 2010; Faguy, 2003). It can be assumed that MTases acquired by archaea from bacteria through horizontal gene transfer likely inherit their functionality (Nölling & de Vos, 1992), but the biological roles of DNA methylation by autochthonous archaeal MTases may differ and remain largely unknown.

The focus of this study was to obtain information on DNA methylation in the genomes of the three halophilic archaea that constitute a xylan‐degrading natural consortium obtained from a hypersaline environment (La Cono et al., 2023). We proposed several biostatistical approaches to gather information on the possible roles of archaeal genome methylation by statistical analysis of the distribution of methylated and unmethylated canonical motifs. The xylanolytic member of the consortium, *Halorabdus* sp. SVX81, emerged as an interesting model for future studies because it possesses complex DNA methylation patterns, most likely associated with active RM systems (Figure 2). However, computational analysis of the distribution of the methylated sites did not allow for prediction of any canonical motifs for this methylation. Similar to certain phage restriction enzymes (Carlson & Kosturko, 1998; Shankar et al., 2017), the SVX81 MTases may recognize rather complex DNA motifs with long spacer regions between protein binding and target sites. In this respect, the motifMaker program used in this study was suboptimal, as it was designed to identify short canonical motifs. The difficulty in identifying canonical motifs prevented the use of this archaeon as a model in the present study.

In contrast, *H. lucertense* SVX82 has proven to be the most useful model for disentangling the possible biological roles of genomic DNA methylation in archaea through the robust statistical analysis of frequencies and patterns of methylated and unmethylated canonical motifs. The SVX82 genome harbours several plasmid‐borne and chromosomal MTases that control the methylation of adenine and cytosine residues at four different canonical DNA motifs. Notably, the methylation patterns at all these motifs were fractional, despite the strain's apparent endonuclease activity (Figure 2). From 20% to 40% of the targeted motifs remained unmethylated for unknown reasons (Table 2). This allowed us to examine the stability of methylation patterns under different growth conditions and identify factors influencing epigenetic modifications of the genomic DNA in this archaeon. Our analysis illuminated that the alternative (lower) GC composition of the target loci is one of the factors underlying the decrease in the efficiency of methylation at canonical motifs in *H. lucertense* SVX82. The integrity of the partially methylated genome can only be maintained if the ability of the cognate REases to bind DNA with alternative GC content is also reduced, considerably diminishing the effectiveness of the RM systems in protecting the host from viruses and conjugative plasmids. The observed inaccessibility of DNA loci with alternative GC compositions to MTases agrees with the published report that the *Haloferax volcanii* chromatin with alternative GC composition is generally less accessible to DNA binding proteins (Marinov et al., 2023). Apparently, for inhabitants of extreme environments, such as many archaea, the possibility of lateral gene exchange is as important for their survival as protection from allochthonous DNA (Wang et al., 2023). Thus, it is highly likely that the reduced efficiency of *H. lucertense* SVX82 RM systems is a trade‐off between the need to protect the cell from intruders, reduce DNA damage due to self‐restriction in stressful environments (Pleška et al., 2016), and the benefits of DNA exchange in the extreme environments.

In this study, we demonstrated a non‐random distribution of the CTAG and GRAGAAG canonical motifs, both methylated and unmethylated, among the protein‐coding regions, regulatory, and non‐coding parts of the *H. lucertense* SVX82 genome, as well as among genes with different expression levels (Figures 6 and 7). Collectively, these findings suggest a rational selection of locations of methylated motifs in the genome sequence associated with certain specific functions. More precisely, the frequency of the GRAGAAG was higher than expected in highly expressed genes of *H. lucertense* SVX82 and these motifs were more likely to be methylated than in lower expressed genes (Supplementary Table S4). The reason for this selection remained unclear but can be associated with higher accessibility of unwound DNA in transcribed regions for the respective MTase.

By contrast, methylation at the *c*TAG motifs did not correlate with gene expression, suggesting a sub‐functionalization of *c*TAG methylation across the genome. The target cytosine residue in *c*TAG motifs was, in most cases, consistently methylated, regardless of gene expression level. This was also true for conditional methylation at these motifs within protein‐coding regions, which did not correlate with the level of gene expression. A commonality observed for many of these genes containing methylated *c*TAG sites was a significant variation in their expression levels, ranging from complete cessation of expression under some conditions to high levels of expression under others (Supplementary Table S3). We hypothesized that *c*TAG methylation in the TSS‐upstream regions could be associated with the accessibility of gene promoter regions to cognate transcription factors (activators and/or inhibitors), thereby affecting the rate of up‐ or down‐regulation. This hypothesis is supported by a previous report (Burgess, 2012). However, our statistical analyses could not conclusively determine whether the growth condition‐dependent changes in methylation patterns of *H. lucertense* SVX82 serve as gene regulation mechanisms or merely reflect changes in chromatin configuration associated with transcriptional regulation. Both hypotheses are equally plausible and require additional study to distinguish between them. Attempts to confirm the involvement of global genome methylation in gene regulation by creating methylation‐deficient mutants have been reported in other studies, yet the results were contradictory. Xu et al. (2021) reported that uropathogenic *E. coli* lacking adenine methylation exhibited significant defects in persister formation during exposure to various antibiotics and stresses, whereas another study by Mehershahi and Chen (2021) reported that knocking out a type I RM system in an *E. coli* strain did not affect gene regulation and the performance of the mutant. Their findings suggest that not all RM systems and global methylation patterns function in a regulatory capacity. The involvement of cytosine methylation in gene regulation has been experimentally confirmed in *E. coli* (Kahramanoglou et al., 2012) and *Vibrio cholerae* (Carvalho et al., 2021). However, no such experiments have been performed on archaea.

The nano‐sized ectosymbiont (<300 nm in diameter), *Ca*. N. occultus SVXNc, has developed intimate interactions with its host, *H. lucertense* SVX82 (La Cono et al., 2023; Reva et al., 2023). Despite its extremely reduced genome, SVXNc contains a huge gene (SVXNc_0300) encoding a giant protein (9409 amino acids) with a possible role in orchestrating specific interactions between the symbiont and its host, presumably serving in the formation of secretion channels (straws) through which the nanohaloarchaea can inject effector(s) favourably affecting the metabolism of the host (Reva et al., 2023). The presence of cytoplasmic bridges of up to 11 nm in diameter was confirmed and visualized using the cryo‐ET technology (Figure 4). The discovery of increased methylation at GDGcHC motifs, which are the canonical methylation motifs of the ectosymbiont (Reva et al., 2023), in the genome of the host archaeon *H. lucertense* SVX82 (Figure 5, Supplementary Figure S4), led us to hypothesize that the MTases might be among the proteins transported from the symbiont to the host cell through this bridge. This transportation of MTase from the symbiont to the host cell may reflect the necessity to protect the host's genomic DNA from the symbiont's REase, whose activity was confirmed experimentally (Figure 2). The persistent, albeit much smaller, methylation of the GDG*c*HC motifs in the genome of *H. lucertense* SVX82 axenic culture (grown without the symbiont) requires explanation in future studies as a possible example of ‘epigenetic memory’ persisting through several generations.

## CONCLUSION

A complex pattern of methylation of genomic nucleotides in *H. lucertense* SVX82 by four endogenous MTases and one MTase transported from the ectosymbiont was discovered. The potential involvement of the ectosymbiont in modifying the host's DNA has never been reported before, adding a new facet to the interactions between haloarchaea and their nanoarchaeal ectosymbionts: namely, the methylation of the host DNA by a methyltransferase that may be imported for the ectosymbiont, or be a way to protect the host DNA from symbiont's endonucleases. This study leveraged statistical approaches to demonstrate the non‐random distribution of methylated and unmethylated canonical motifs controlled by different MTases in *H. lucertense* SVX82. The preferential appearance of methylated sites in the TSS‐upstream regions and in genes characterized by elevated expression suggests a possible involvement of methylation of genomic nucleotides in gene regulation.

These discoveries make *H. lucertense* SVX82 a valuable model archaeon for future studies on the biological roles of genome methylation in archaea—an area of research that remains generally understudied. To the best of our knowledge, our study is the first to investigate methylation patterns in archaeal communities and to statistically validate variations in genome methylation depending on the structure of the archaeal consortium.

## AUTHOR CONTRIBUTIONS

**Oleg N. Reva:** Conceptualization (equal); data curation (equal); formal analysis (equal); investigation (equal); methodology (equal); software (equal); visualization (equal); writing – original draft (equal); writing – review and editing (equal). **Violetta La Cono:** Data curation (equal); investigation (equal); methodology (equal); writing – review and editing (supporting). **Francesca Crisafi:** Data curation (equal); formal analysis (equal); methodology (equal); writing – review and editing (supporting). **Francesco Smedile:** Data curation (equal); formal analysis (equal); methodology (equal); software (equal); writing – review and editing (supporting). **Manasi Mudaliyar:** Data curation (equal); formal analysis (equal); investigation (equal); methodology (equal); software (equal); writing – review and editing (equal). **Debnath Ghosal:** Funding acquisition (equal); investigation (equal); methodology (equal); supervision (equal); writing – review and editing (equal). **Laura Giuliano:** Conceptualization (equal); formal analysis (equal); validation (equal); writing – review and editing (equal). **Mart Krupovic:** Formal analysis (equal); software (equal); writing – review and editing (equal). **Michail M. Yakimov:** Conceptualization (equal); data curation (equal); formal analysis (equal); funding acquisition (equal); investigation (equal); methodology (equal); resources (equal); supervision (equal); validation (equal); visualization (equal); writing – original draft (equal); writing – review and editing (equal).

## CONFLICT OF INTEREST STATEMENT

The authors declare no conflicts of interest.

## Supporting information


**Supplementary Figure S1.** Atlas view of the genome of *H. lucertense* SVX82g composed of the chromosome and the three plasmids shown as brown and dark‐green arcs. Genomic islands identified by the SeqWord Genome Island Sniffer and the respective metrics: GC content; the ratio of generalized relative variance (GRV) versus relative variance (RV) of distribution of nucleotide tetramers normalized by GC content (n1_4mer); and distance D between local and global tetramer frequency patterns calculated in a 5 kbp sliding window stepping 2 kbp used for detection of genomic islands (see http://seqword.bi.up.ac.za/sniffer/index.html for more detail) are shown respectively by pink blocks and coloured histograms as explained in the legend. Locations of methyltransferase (MT) and restriction endonuclease (RE) genes are depicted by triangle red marks.

**Supplementary Figure S2.** Venn diagrams, contingency tables, and estimated Chi2 metrics visualize the distribution of unmethylated CTAG motifs on the chromosome (A) and the plasmids (B); and unmethylated CCTTG motifs on the chromosome (C) and the plasmids (D) of *H. lucertense* SVX82 in different experiments: (I) pure (axenic) culture on d‐xylose; (II) binary culture with the ectosymbiont Ca. N. occultus SVXNc on d‐xylose; (III) binary culture with *Halorabdus* sp. SVX81 on xylan; (IV) trinary culture with *Halorabdus* sp. SVX81 and the ectosymbiont Ca. N. occultus SVXNc on xylan.

**Supplementary Figure S3.** Venn diagrams, contingency tables, and estimated Chi2 metrics visualize the distribution of unmethylated GTCGAGG motifs on the chromosome (A) and the plasmids (B); and unmethylated GRAGAAG motifs on the chromosome (C) and the plasmids (D) of *H. lucertense* SVX82 in different experiments: (I) pure (axenic) culture on d‐xylose; (II) binary culture with the ectosymbiont Ca. N. occultus SVXNc on d‐xylose; (III) binary culture with *Halorabdus* sp. SVX81 on xylan; (IV) trinary culture with *Halorabdus* sp. SVX81 and the ectosymbiont Ca. N. occultus SVXNc on xylan.

**Supplementary Figure S4.** Venn diagrams, contingency tables, and estimated Chi2 metrics visualize the distribution of methylated GDG*c*HC motifs on the chromosome (A) and the plasmids (B) of *H. lucertense* SVX82 in different experiments: (I) pure (axenic) culture on d‐xylose; (II) binary culture with the ectosymbiont Ca. N. occultus SVXNc on d‐xylose; (III) binary culture with *Halorabdus* sp. SVX81 on xylan; (IV) trinary culture with *Halorabdus* sp. SVX81 and the ectosymbiont Ca. N. occultus SVXNc on xylan.

**Supplementary Table S1.** Composition of consortia used in this study (120 h of cultivation).

**Supplementary Table S2.** SRA NCBI database accession numbers of the raw SMRT PacBio genomic reads and Illumina RNA reads generated for this study.

**Supplementary Table S3.** Protein coding genes H. lucertense SVX82 with cTAG methylation within 20 bp upstream of the start codon.

**Supplementary Table S4.** Methylation and expression of genes with multiple GRAGa G methylation motifs within their sequences.

**Supplementary Video S1.** Three‐dimensional (segmented) view of host (SVX82, maroon outer layer) and DPANN (SVXNc, cyan outer layer) interaction showing extensive membrane blebbing from the DPANN SVXNc at the DPANN‐host interface.

## Data Availability

In‐house software tool SeqWord MotifMapper 3.1 developed for this study for visualization and analysis of methylation profiles supplemented with all input files: GBK genomes and GFF files generated by the program ipdSummary for *H. lucertense* and its symbiont, containing genome methylation data at different growth conditions: https://zenodo.org/doi/10.5281/zenodo.10700787. Accession numbers in the NCBI repository: Whole genome sequences: *Halorhabdus* sp. SVX81 – CP104322; *H. lucertense* SVX82 – CP104741‐44; and Ca. Na. occultus SVXNc – CP104395; PacBio raw read data: Archaeal consortium I, PacBio Exp 1.1 – SRX22141242; Archaeal consortium I, PacBio Exp 1.2 – SRX22141245; Archaeal consortium II, PacBio Exp 2.1 – SRX22141234; Archaeal consortium II, PacBio Exp 2.2 – SRX22141235; Archaeal consortium III, PacBio Exp 3.1 – SRX22141243; Archaeal consortium III, PacBio Exp 3.2 – SRX22141244; Archaeal consortium IV, PacBio Exp 4 – SRX22141246; RNA‐seq data: Archaeal consortium I, Illumina‐RNA Exp 1.1 – SRX22141247; Archaeal consortium I, Illumina‐RNA Exp 1.2 – SRX22141248; Archaeal consortium II, Illumina‐RNA Exp 2.1 – SRX22141249; Archaeal consortium I, Illumina‐RNA Exp 2.2 – SRX22141236; Archaeal consortium III, Illumina‐RNA Exp 3.1 – SRX22141237; Archaeal consortium III, Illumina‐RNA Exp 3.2 – SRX22141238; Archaeal consortium IV; Archaeal consortium IV, Illumina‐RNA Exp 4.1 – SRX22141239; Archaeal consortium IV, Illumina‐RNA Exp 4.2 – SRX22141240; Illumina‐RNA Exp 4.3 – SRX22141241; PacBio methylation data: *H. lucentense* Exp.I – SUPPF_0000005533; *H. lucentense* Exp.II – SUPPF_0000005534; *H. lucentense* Exp.III – SUPPF_0000005535; *H. lucentense* Exp.IV – SUPPF_0000005536; Ca. Nanohaloarchaeota archaeon SVXNc Exp.II – SUPPF_0000005537; Ca. Nanohaloarchaeota archaeon SVXNc Exp.IV – SUPPF_0000005538.
