# Supplementary figures and images for: Interplay of intracellular and trans‐cellular DNA methylation in natural archaeal consortia
Source: Environ Microbiol Rep. 2024 Apr 8;16(2):e13258. doi: 10.1111/1758-2229.13258 (PMC11001535; doi:10.1111/1758-2229.13258)

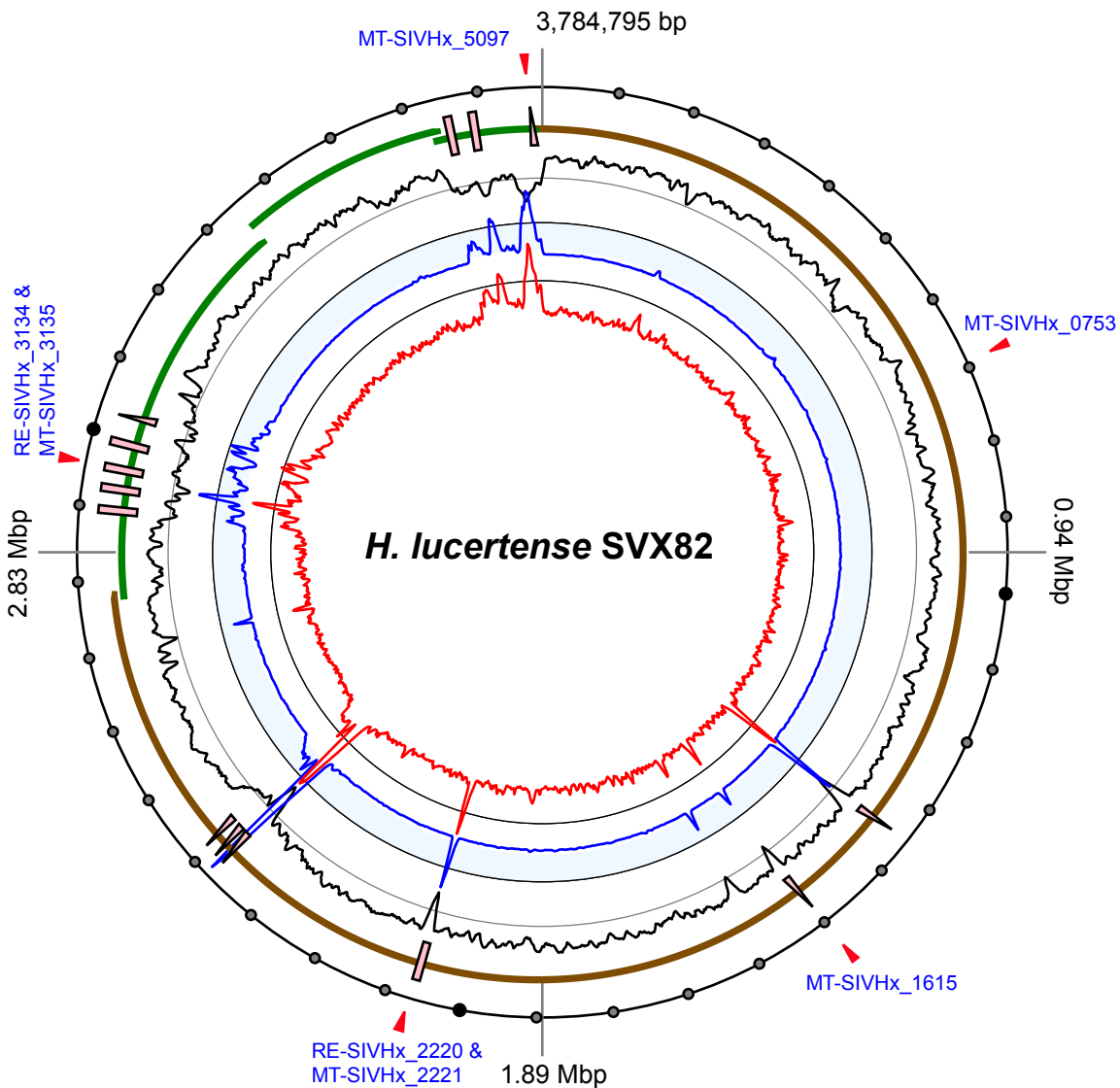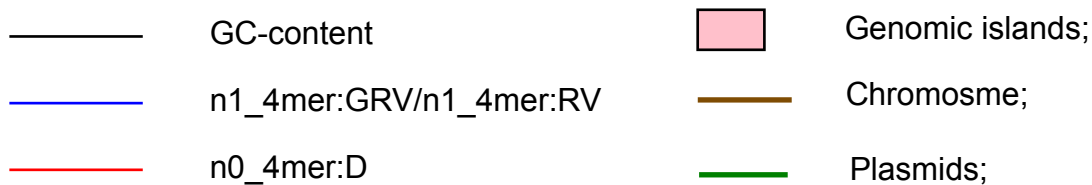

Supplement: Supplementary file 1 — Supplementary Figure S1. Atlas view of the genome of H. lucertense SVX82g composed of the chromosome and the three plasmids shown as brown and dark‐green arcs. Genomic islands identified by the SeqWord Genome Island Sniffer and the respective metrics: GC content; the ratio of generalized relative variance (GRV) versus relative variance (RV) of distribution of nucleotide tetramers normalized by GC content (n1_4mer); and distance D between local and global tetramer frequency patterns calculated in a 5 kbp sliding window stepping 2 kbp used for detection of genomic islands (see http://seqword.bi.up.ac.za/sniffer/index.html for more detail) are shown respectively by pink blocks and coloured histograms as explained in the legend. Locations of methyltransferase (MT) and restriction endonuclease (RE) genes are depicted by triangle red marks. [file EMI4-16-e13258-s008.pdf]

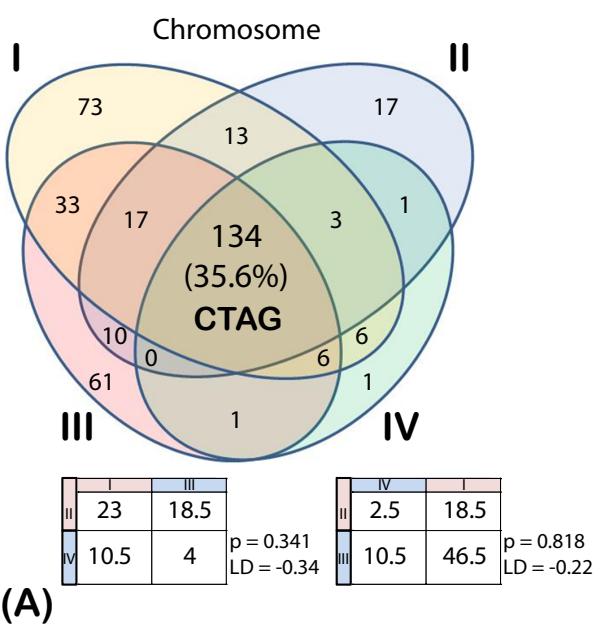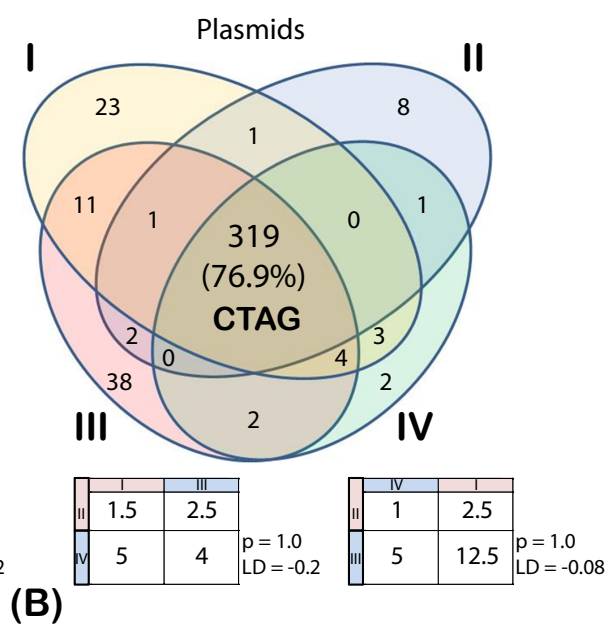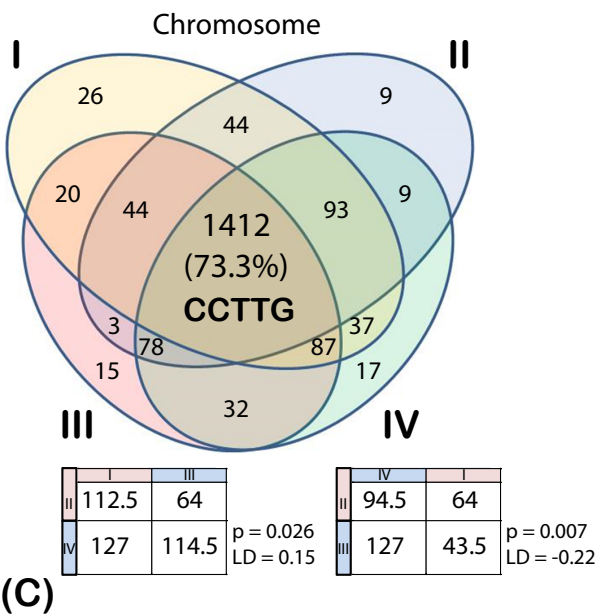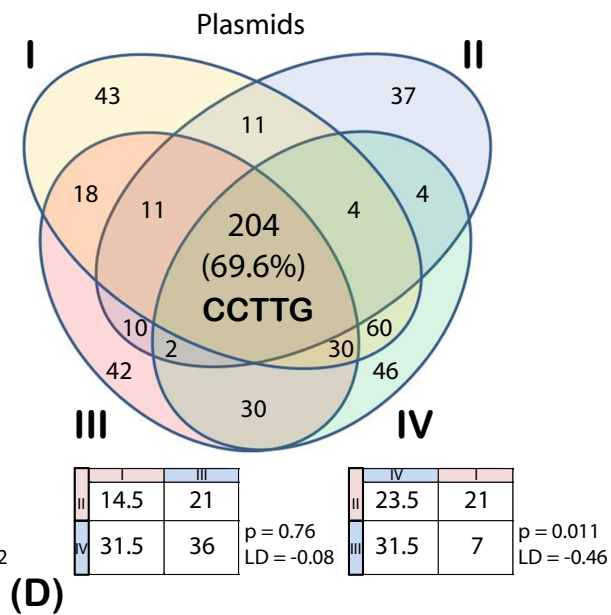

Supplement: Supplementary file 2 — Supplementary Figure S2. Venn diagrams, contingency tables, and estimated Chi2 metrics visualize the distribution of unmethylated CTAG motifs on the chromosome (A) and the plasmids (B); and unmethylated CCTTG motifs on the chromosome (C) and the plasmids (D) of H. lucertense SVX82 in different experiments: (I) pure (axenic) culture on d‐xylose; (II) binary culture with the ectosymbiont Ca. N. occultus SVXNc on d‐xylose; (III) binary culture with Halorabdus sp. SVX81 on xylan; (IV) trinary culture with Halorabdus sp. SVX81 and the ectosymbiont Ca. N. occultus SVXNc on xylan. [file EMI4-16-e13258-s007.pdf]

# Chromosome

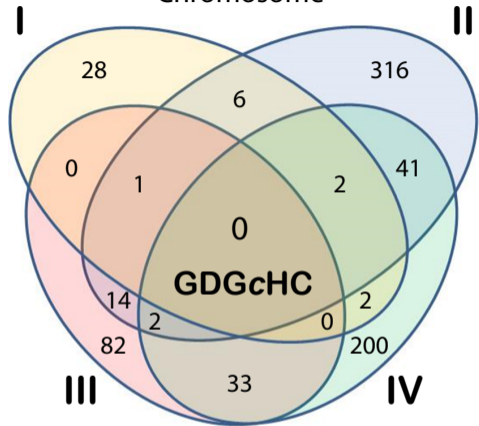

|    | II  | III  |
|----|-----|------|
| II | 7.5 | 15.5 |
| IV | 3   | 34   |

$p = 0.031$   
LD = 0.55

|     | IV | III  |
|-----|----|------|
| II  | 45 | 15.5 |
| III | 3  | 7.5  |

$p = 0.008$   
LD = 0.59

(A)

# Plasmids

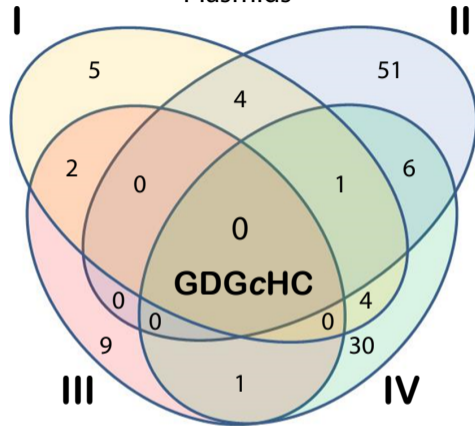

|    | II  | III |
|----|-----|-----|
| II | 5.5 | 0   |
| IV | 5.5 | 1   |

$p = 1.0$   
LD = 1.0

|     | IV  | III |
|-----|-----|-----|
| II  | 7.5 | 0   |
| III | 5.5 | 2   |

$p = 0.45$   
LD = 1.0

(B)

Supplement: Supplementary file 4 — Supplementary Figure S4. Venn diagrams, contingency tables, and estimated Chi2 metrics visualize the distribution of methylated GDGcHC motifs on the chromosome (A) and the plasmids (B) of H. lucertense SVX82 in different experiments: (I) pure (axenic) culture on d‐xylose; (II) binary culture with the ectosymbiont Ca. N. occultus SVXNc on d‐xylose; (III) binary culture with Halorabdus sp. SVX81 on xylan; (IV) trinary culture with Halorabdus sp. SVX81 and the ectosymbiont Ca. N. occultus SVXNc on xylan. [file EMI4-16-e13258-s004.pdf]
