# Supplementary material for: Interplay of intracellular and trans‐cellular DNA methylation in natural archaeal consortia
Source: Environ Microbiol Rep. 2024 Apr 8;16(2):e13258. doi: 10.1111/1758-2229.13258 (PMC11001535; doi:10.1111/1758-2229.13258)
Supplement: Supplementary file 3 — Supplementary Figure S3. Venn diagrams, contingency tables, and estimated Chi2 metrics visualize the distribution of unmethylated GTCGAGG motifs on the chromosome (A) and the plasmids (B); and unmethylated GRAGAAG motifs on the chromosome (C) and the plasmids (D) of H. lucertense SVX82 in different experiments: (I) pure (axenic) culture on d‐xylose; (II) binary culture with the ectosymbiont Ca. N. occultus SVXNc on d‐xylose; (III) binary culture with Halorabdus sp. SVX81 on xylan; (IV) trinary culture with Halorabdus sp. SVX81 and the ectosymbiont Ca. N. occultus SVXNc on xylan. [file EMI4-16-e13258-s003.pdf]

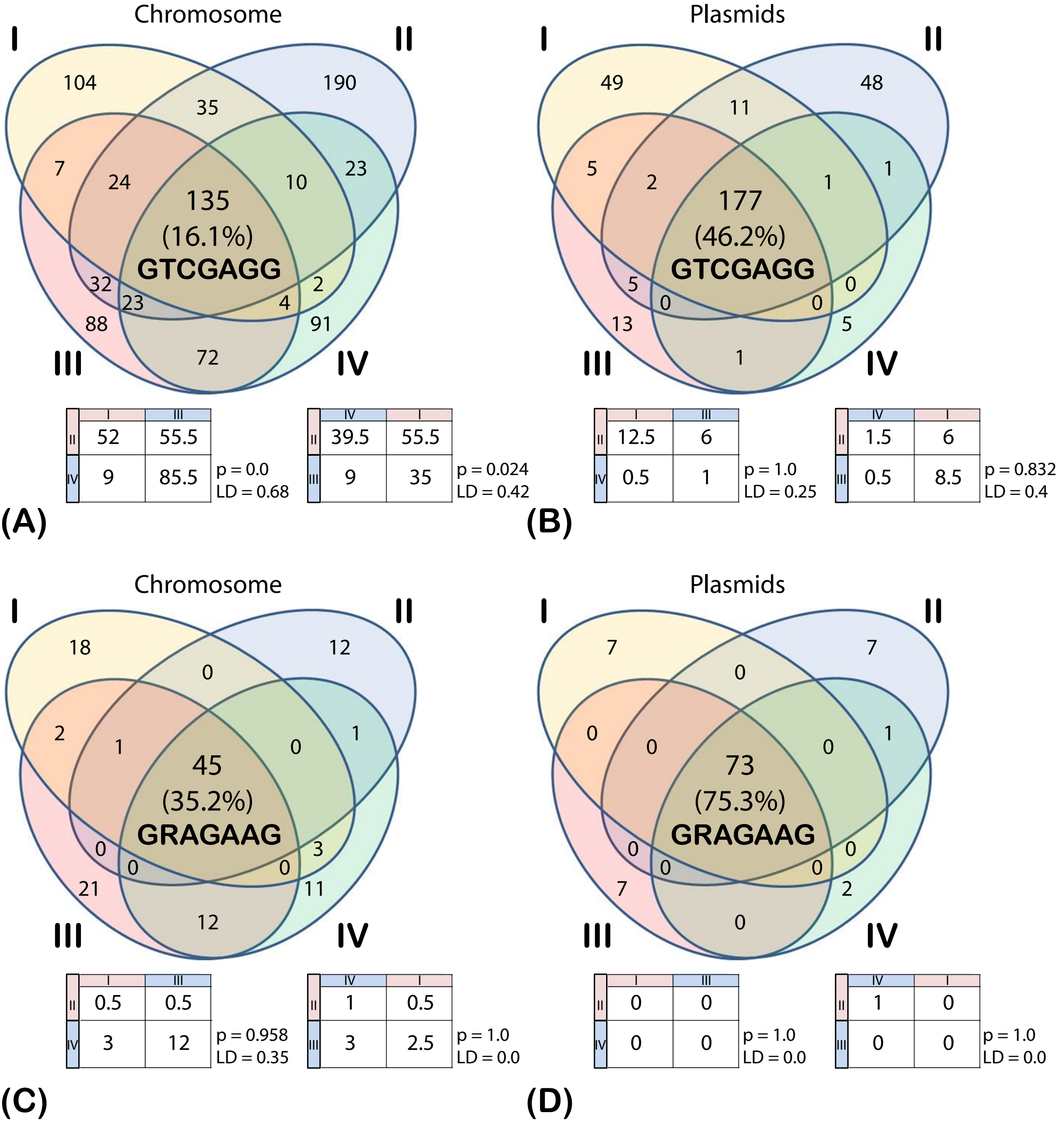
Chromosome
I
II
III
IV
104
35
190
7
24
135
(16.1%)
GTCGAGG
10
23
32
23
4
2
88
72
91
I
III
II
52
55.5
IV
9
85.5
p = 0.0
LD = 0.68
IV
I
II
39.5
55.5
III
9
35
p = 0.024
LD = 0.42
(A)
Plasmids
I
II
III
IV
49
11
48
5
2
177
(46.2%)
GTCGAGG
1
1
5
0
0
0
13
1
5
I
III
II
12.5
6
IV
0.5
1
p = 1.0
LD = 0.25
IV
I
II
1.5
6
III
0.5
8.5
p = 0.832
LD = 0.4
(B)
Chromosome
I
II
III
IV
18
0
12
2
1
45
(35.2%)
GRAGAAG
0
1
0
0
0
3
21
12
11
I
III
II
0.5
0.5
IV
3
12
p = 0.958
LD = 0.35
IV
I
II
1
0.5
III
3
2.5
p = 1.0
LD = 0.0
(C)
Plasmids
I
II
III
IV
7
0
7
0
0
73
(75.3%)
GRAGAAG
0
1
0
0
0
0
7
0
2
I
III
II
0
0
IV
0
0
p = 1.0
LD = 0.0
IV
I
II
1
0
III
0
0
p = 1.0
LD = 0.0
(D)
