# Supplementary material for: Interplay of intracellular and trans‐cellular DNA methylation in natural archaeal consortia
Source: Environ Microbiol Rep. 2024 Apr 8;16(2):e13258. doi: 10.1111/1758-2229.13258 (PMC11001535; doi:10.1111/1758-2229.13258)
Supplement: Supplementary file 5 — Supplementary Table S1. Composition of consortia used in this study (120 h of cultivation). [file EMI4-16-e13258-s005.pdf]

Suppl. Table S1. Composition of consortia used in this study (120 hours of cultivation).

Consortia	Source of carbon and energy (g L^{-1})	Composition*	Depth of aligned PacBio reads	Cell density, x 10^7 mL^{-1}
I<br>ISP-PB6,9	10 mM D-xylose (1.5)	*H. lucertense* SVX82 – 100%	77	31.6 ± 6.14†
II<br>ISP-PB1,2	10mM D-xylose (1.5)	*H. lucertense* SVX82 – 33%<br>*Ca.* N. occultus SVXNc – 67%	161<br>374	11.2 ± 1.45†<br>31.9 ± 4.57‡
III<br>ISP-PB7,8	Beechwood xylan (1.5 g)	*H. lucertense* SVX82 – 17%<br>*Halorhabdus* sp. SVX81 – 83%	57<br>476	3.51 ± 0.88†<br>10.6 ± 3.17†
IV<br>SX3aSN	Beechwood xylan (1.5 g)	*H. lucertense* SVX82 – 11%<br>*Ca.* N. occultus SVXNc – 34%<br>*Halorhabdus* sp. SVX81 – 55%	164<br>779<br>1444	2.31 ± 0.68†<br>7.18 ± 2.12‡<br>4.62 ± 1.97†

*Proportion of archaeal strains in the consortia were identified by comparison of mean depth values of long PacBio reads aligned against the reference whole genome sequences of these strains estimated by Samtools-1.10;

†Cell density for haloarchaea were calculated as described previously (La Cono et al., 2023). Briefly, at 120h of incubation at 40°C statically, grown cultures were resuspended and 100 μL was diluted to 10^{-5} – 10^{-7} and 20–50 μL of these 10-fold serial dilutions were plated as five spatially separated droplets on LC agar (1.5%, w/v) plates supplemented with D-xylose (30 mM). Grown colonies originated on the Petri dishes from a single cell were counted as cells mL^{-1};

‡quantitative PCR (qPCR) method was used to determine the relative cell densities of *Ca*. Nanohalococcus occultus as previously described (La Cono et al., 2020).
