# Supplementary material for: Interplay of intracellular and trans‐cellular DNA methylation in natural archaeal consortia
Source: Environ Microbiol Rep. 2024 Apr 8;16(2):e13258. doi: 10.1111/1758-2229.13258 (PMC11001535; doi:10.1111/1758-2229.13258)
Supplement: Supplementary file 6 — Supplementary Table S2. SRA NCBI database accession numbers of the raw SMRT PacBio genomic reads and Illumina RNA reads generated for this study. [file EMI4-16-e13258-s009.pdf]

Suppl. Table S2. SRA NCBI database accession numbers of the raw SMRT PacBio genomic reads and Illumina RNA reads generated for this study.

**Consortium**	**Sample name**	**Number of reads**	**Accession number**
**SMRT PacBio reads**			
**I**	ISP-PB6a	34,376	SRX22141242
	ISP-PB6b	36,290	
	ISP-PB9	21,184	SRX22141245
**II**	ISP-PB1	38,824	SRX22141234
	ISP-PB2	132,203	SRX22141235
**III**	ISP-PB7	98,688	SRX22141243
	ISP-PB8	139,929	SRX22141244
**IV**	SX3aSN	1,085,643	SRX22141246
**Illumina HiSeq RNA reads**			
**I**	5_XS_S6	23,511,982	SRX22141247
	SX83a2_XS3	20,164,144	SRX22141248
**II**	4a_S1	25,347,949	SRX22141249
	4a_S2	24,042,256	SRX22141236
**III**	S_SX82-1	21,807,457	SRX22141237
	S_SX82-2	18,522,458	SRX22141238
**IV**	SX3ASN	19,204,142	SRX22141239
	SX83a_BN5d	21,650,662	SRX22141240
	SX83a_SN10d	22,735,729	SRX22141241
