# Supplementary material for: Interplay of intracellular and trans‐cellular DNA methylation in natural archaeal consortia
Source: Environ Microbiol Rep. 2024 Apr 8;16(2):e13258. doi: 10.1111/1758-2229.13258 (PMC11001535; doi:10.1111/1758-2229.13258)
Supplement: Supplementary file 7 — Supplementary Table S3. Protein coding genes H. lucertense SVX82 with cTAG methylation within 20 bp upstream of the start codon. [file EMI4-16-e13258-s006.pdf]

Suppl. Table S3. Protein coding genes *H. lucertense* SVX82 with cTAG methylation within 20 bp upstream of the start codon

Locus Tag	Δ*	Methylation†				RPKM‡				Gene annotation
		I	II	III	IV	I	II	III	IV	
SIVHX_0538	-3	+	+	+	+	44	0	44	0	HAD superfamily hydrolase
SIVHX_2584	-4	+	+	+	+	82	47	0	62	Sugar-specific transcriptional regulator TrmB
SIVHX_0106	-5	+	+	+	+	70	73	0	0	Uncharacterized protein
SIVHX_0772	-5	--	+	+	+	238	217.5	47	147	CbpA; DnaJ-class molecular chaperone
SIVHX_1729	-5	+	+	+	+	37.5	13.5	59	31	Uncharacterized membrane protein, Yip1 family
SIVHX_1861	-5	+	+	+	+	813.5	585.5	121	541.5	Ndk; Nucleoside diphosphate kinase
SIVHX_2482	-5	--	+	+	+	21	19.5	25	0	BglX; Beta-glucosidase, glycosyl hydrolase family 3
SIVHX_3282	-5	+	+	+	+	0	23	0	0	Uncharacterized protein
SIVHX_0549	-6	+	--	+	+	1229.5	707.5	267.5	821.5	Predicted redox protein, regulator of disulfide bond formation
SIVHX_2487	-6	+	+	+	+	202.5	275.5	230	351.5	SalX; ABC-type antimicrobial peptide transport system
SIVHX_3146	-6	+	+	+	+	95	151	96	100.5	OmpR; REC domain
SIVHX_5036	-8	+	+	--	+	183.5	285	0	187.5	ArsR; Transcriptional regulator containing HTH domain, ArsR family
SIVHX_0132	-9	+	+	+	+	135.5	149	32	42.5	RIO1; Serine/threonine protein kinase involved in cell cycle control
SIVHX_1326	-9	--	+	+	--	429.5	510.5	74	348.5	Transcriptional regulator, contains HTH domain
SIVHX_2851	-9	+	--	+	+	259.5	289.5	586.5	254	CopA; Copper-translocating P-type ATPase
SIVHX_0064	-10	--	+	+	+	817.5	540	346	598	Rubredoxin family protein
SIVHX_0624	-10	--	+	+	+	716	1950.5	38.5	2863.5	THI4; Sulfide-dependent adenosine diphosphate thiazole synthase
SIVHX_0972	-11	+	+	+	+	123	65.5	70.5	182.5	Archaea-specific RecJ-like exonuclease
SIVHX_1140	-11	+	+	+	--	1056	537.5	561.5	3172	AldH; Aldehyde dehydrogenase (NAD+)
SIVHX_2840	-11	+	+	+	+	311	179	285.5	229.5	Fdx; DnaJ-like chaperone fused to ferredoxin
SIVHX_0241	-12	+	+	+	+	136.5	27	108.5	121	ICC-like phosphoesterase
SIVHX_0476	-12	--	+	+	+	25.5	32	0	36.5	Uncharacterized protein
SIVHX_0761	-12	+	+	--	+	72.5	50	330.5	197	Membrane protein implicated in regulation of membrane protease activity
SIVHX_1096	-12	+	+	+	+	62	0	129.5	0	Uncharacterized protein
SIVHX_1193	-12	--	+	+	+	143.5	113	279	344.5	Twin-arginine translocation signal domain-containing protein
SIVHX_1769	-12	+	+	+	+	55.5	36.5	0	0	Uncharacterized protein
SIVHX_2246	-12	+	+	--	+	91	107.5	0	0	Uncharacterized protein
SIVHX_2274	-12	--	+	--	+	20	25	96.5	28	AcrR; Transcriptional regulator, TetR/AcrR family
SIVHX_2438	-12	+	+	+	+	31.5	18.5	189.5	0	Cytochrome cbb3 oxidase subunit I
SIVHX_2809	-12	--	+	+	+	95	104	0	43.5	Uncharacterized protein
SIVHX_4082	-12	--	+	+	+	301.5	89.5	96	75	NosL family protein
SIVHX_0095	-13	+	+	+	+	104.5	131	56.5	0	NMD3; NMD protein affecting ribosome stability and mRNA decay
SIVHX_0302	-13	+	+	+	+	381.5	398	267	511.5	eRF1; Peptide chain release factor eRF1
SIVHX_0432	-13	+	+	+	+	205	300.5	1091.5	295	Uncharacterized archaeal Zn-finger protein
SIVHX_0545	-13	+	+	+	+	51.5	8	0	142.5	HisC; Histidinol-phosphate aminotransferase
SIVHX_1192	-13	--	+	+	+	524	501	232	433.5	Sco1; Cytochrome oxidase Cu insertion factor, SCO1/SenC/PrrC family
SIVHX_1297	-13	+	+	+	+	116.5	208	0	139.5	ArsR; Transcriptional regulator containing HTH domain, ArsR family
SIVHX_1533	-13	+	+	+	+	80.5	119	0	32	ArgA; Acetyltransferase (GNAT) family
SIVHX_1600	-13	+	+	--	+	40.5	87	280.5	246.5	Zn finger protein, C2C2 type
SIVHX_1767	-13	+	+	+	+	132.5	167.5	106	177	AstE; Succinylglutamate desuccinylase
SIVHX_2142	-13	+	+	--	+	90	142	125	374	Uncharacterized protein, DUF2062 family
SIVHX_2574	-13	--	+	--	+	6.5	0	0	0	FepB; ABC-type Fe3+-hydroxamate transport system
SIVHX_2595	-13	+	+	+	+	67.5	13.5	0	72.5	Uncharacterized protein
SIVHX_4056	-13	+	+	+	+	0	0	0	0	FrdA; Fumarate reductase flavoprotein subunit
SIVHX_0267	-14	+	+	+	+	11.5	27	0	53.5	CinA; Nucleotide-utilizing enzyme related to molybdopterin-biosynthesis
SIVHX_1073	-14	+	--	--	+	509.5	559.5	421.5	420.5	PetE; Halocyanin
SIVHX_1520	-14	--	+	+	--	146	175	321	536	GyrB; DNA gyrase subunit B
SIVHX_2227	-14	+	--	+	+	5	6	0	0	Uncharacterized surface protein, possible component of pili like system
SIVHX_1137	-15	+	+	--	+	1624	3529	1263	1974	Uncharacterized protein
SIVHX_2494	-16	+	+	+	+	41.5	112.5	17.5	51.5	Uncharacterized protein with Ig-like domain
SIVHX_3150	-16	+	+	+	+	50.5	65.5	0	0	hypothetical protein
SIVHX_3282	-16	+	+	+	+	0	23	0	0	Uncharacterized protein
SIVHX_4004	-16	+	+	+	+	1475	2009	145	7173.5	Pilin/Flagellin, FlaG/FlaF family
SIVHX_1072	-17	--	+	+	+	666.5	742	209	554.5	Zn ribbon containing protein
SIVHX_3327	-17	+	+	+	+	58	56.5	0	45	SufS; Cysteine desulfurase/selenocysteine lyase
SIVHX_0202	-18	+	+	--	+	33.5	116.5	0	49	Predicted trancriptional regulator, ArsR family
SIVHX_0279	-18	+	+	+	+	403	541.5	3387	540	Predicted RNA-binding protein, contains TRAM domain
SIVHX_1893	-18	+	+	+	+	236	71.5	29	38.5	IolE; Sugar phosphate isomerase/epimerase
SIVHX_3284	-18	+	+	+	+	36.5	91.5	0	0	Transcriptional regulator, contains HTH domain
SIVHX_3288	-18	+	+	+	+	30	66.5	23	30.5	Protein containing two CBS domains
SIVHX_0562	-19	+	+	+	+	28	81	0	21	Transposase
SIVHX_2215	-19	+	+	+	+	4	24.5	0	0	Transposase
SIVHX_3124	-19	--	+	--	+	7	15.5	0	0	Transposase
SIVHX_5067	-19	+	+	+	+	163.5	246.5	251	0	Transcriptional regulator, PadR family
SIVHX_0522	-20	+	+	+	+	77	34	60	77	Two-component system, OmpR family, sensor kinase
SIVHX_1115	-20	+	+	--	+	1454	2612.5	807	1201.5	TatA; Sec-independent protein secretion pathway component
SIVHX_3021	-20	+	+	+	+	7.5	24.5	0	0	Transposase
SIVHX_3300	-20	--	+	+	+	215	66	71.5	21	Transposase

*Distance from the methylated cytosine to the start codon;

†Methylation occurrence in four experiments I, II, III and IV.

‡RPKM (reads per kilo base per million mapped reads) values of gene expression in four experiments I, II, III and IV.
