# Supplementary material for: Interplay of intracellular and trans‐cellular DNA methylation in natural archaeal consortia
Source: Environ Microbiol Rep. 2024 Apr 8;16(2):e13258. doi: 10.1111/1758-2229.13258 (PMC11001535; doi:10.1111/1758-2229.13258)
Supplement: Supplementary file 8 — Supplementary Table S4. Methylation and expression of genes with multiple GRAGa G methylation motifs within their sequences. [file EMI4-16-e13258-s001.pdf]

Suppl. Table S4. Methylation and expression of genes with multiple GRAG*a*G methylation motifs within their sequences.

Locus tag	N*	Methylated sites in different experiments†				RPKM‡ values per experiment and the average expression					Annotation
		I	II	III	IV	I	II	III	IV	Average	
SIVHX_0300	5	5	5	5	5	824	1028.5	9855	1305	3253.1	AtpD; Archaeal/vacuolar-type H+-ATPase subunit D
SIVHX_1454	4	4	4	2	2	768.5	2478.5	9987	1125	3589.8	LeuC; 3-isopropylmalate dehydratase large subunit
SIVHX_1879	3	3	2	3	3	1168	2455.5	202	1346.5	1293.0	RPL37A; Ribosomal protein L37E
SIVHX_0328	3	3	3	0	0	769.5	1599	863	2092	1330.9	RpoB2; DNA-directed RNA polymerase subunit B''
SIVHX_1140	3	3	3	3	3	1056	537.5	561.5	3172	1331.8	AldH; Aldehyde dehydrogenase (NAD+)
SIVHX_0115	3	3	3	3	3	316.5	397	530	430.5	418.5	FtsY; Signal recognition particle GTPase
SIVHX_1653	3	3	3	3	3	224	201	305.5	133	215.9	GlpC; Fe-S oxidoreductase
SIVHX_1640	3	3	3	3	3	193.5	224	47.5	281	186.5	ThrS; Threonyl-tRNA synthetase
SIVHX_1802	3	3	3	3	3	137.5	83	32.5	94	86.8	LivG; ABC-type branched-chain amino acid transport system, ATPase component
SIVHX_2072	3	3	3	3	3	120.5	78	79.5	46	81.0	CrtI; Phytoene desaturase
SIVHX_0851	3	2	3	3	3	54.5	54.5	17	15.5	35.4	CaiC; Acyl-CoA synthetase (AMP-forming)/AMP-acid ligase II
SIVHX_0660	2	1	2	2	2	2921	4557	7423.5	3797.5	4674.8	RPL42A; Ribosomal protein L44E
SIVHX_0760	2	2	2	1	1	1354	1286	5853	2489.5	2745.6	HflC; Membrane protease subunit, stomatin/prohibitin-like protein
SIVHX_0808	2	2	2	2	2	2000.5	1968.5	3072.5	1686.5	2182.0	RPT1; ATP-dependent 26S proteasome regulatory subunit
SIVHX_0410	2	2	2	2	2	1144	1141	2720	1921	1731.5	GroEL; Chaperonin GroEL, HSP60 family
SIVHX_0330	2	2	2	1	1	788.5	1289.5	2569.5	2199	1711.6	RpoA; DNA-directed RNA polymerase subunit A'
SIVHX_2047	2	2	2	2	2	1482	1480	2315	417	1423.5	RPS4A; Ribosomal protein S4E
SIVHX_2042	2	2	2	2	2	1937	1162	795.5	328	1055.6	RpmC; Ribosomal protein L29
SIVHX_0235	2	2	2	2	1	1307	926.5	1220	995.5	1112.3	GlnA; Glutamine synthetase
SIVHX_0329	2	2	2	0	0	726.5	1065	993.5	1708.5	1123.4	RpoB; DNA-directed RNA polymerase subunit B'
SIVHX_1877	2	2	2	2	1	827	920	382	2366.5	1123.9	Rnj; Ribonuclease J
SIVHX_0831	2	2	2	1	1	393.5	490.5	1355.5	1327	891.6	Beta-CASP ribonuclease aCPSF1
SIVHX_1132	2	2	2	2	2	43.5	119	0	68	57.6	Gamma-carboxymuconolactone-like protein, decarboxylase subunit
SIVHX_2017	2	2	2	2	2	1244.5	1033	404.5	824.5	876.6	NadA; Quinolinate synthase
SIVHX_0291	2	2	2	2	2	965.5	1163.5	361	517.5	751.9	AtpH; Archaeal/vacuolar-type H+-ATPase subunit H
SIVHX_2107	2	2	2	2	2	719.5	578.5	773	565	659.0	DUF106 domain-containing protein
SIVHX_1721	2	2	2	2	2	630.5	318	837	937	680.6	FumC; Fumarate hydratase, class II
SIVHX_0494	2	2	2	2	2	471	564	656	869.5	640.1	AcnA; Aconitate hydratase
SIVHX_2038	2	2	2	2	2	935	447.5	479	603.5	616.3	RplB; Ribosomal protein L2
SIVHX_1077	2	2	2	2	2	440	349.5	792	244.5	456.5	TrxB; Thioredoxin reductase (NADPH)
SIVHX_1492	2	2	2	2	2	597	251.5	199.5	600	412.0	DhaK; Dihydroxyacetone kinase subunit DhaK
SIVHX_0118	2	2	2	2	2	380	502	152	378.5	353.1	Ffh; Signal recognition particle GTPase
SIVHX_0643	2	2	2	2	2	224.5	206.5	552.5	181	291.1	GatB; Aspartyl-tRNA(Asn)/Glutamyl-tRNA(Gln) amidotransferase subunit
SIVHX_0648	2	2	2	2	2	176.5	176	518.5	358	307.3	Smc; Chromosome segregation protein SMC
SIVHX_0737	2	2	2	2	2	118.5	73.5	743	234.5	292.4	GroEL; Chaperonin GroEL, HSP60 family
SIVHX_2023	2	2	2	2	2	248.5	261	159	302	242.6	GTP-binding protein
SIVHX_1787	2	2	2	2	2	511.5	259.5	46	205.5	255.6	FadB; 3-hydroxyacyl-CoA dehydrogenase/Enoyl-CoA hydratase
SIVHX_1890	2	2	2	2	2	79.5	30	584.5	236.5	232.6	RtcB; RNA 3'-P ligase, RtcB family protein
SIVHX_2931	2	2	2	2	2	229	309.5	129	281.5	237.3	MPH1; ERCC4-like helicase
SIVHX_2696	2	2	2	2	2	102.5	465.5	241.5	0	202.4	Tsr3; Ribosome biogenesis protein Tsr3
SIVHX_2877	2	2	2	2	2	207.5	273	0	442.5	230.8	PdhA; Pyruvate/2-oxoglutarate dehydrogenase complex, dehydrogenase (E1) component, alpha subunit
SIVHX_0761	2	2	2	2	2	72.5	50	330.5	197	162.5	Membrane protein implicated in regulation of membrane protease activity
SIVHX_1262	2	2	2	2	2	140.5	162	83.5	387.5	193.4	HerA helicase
SIVHX_3147	2	2	2	2	2	155.5	235	21.5	209	155.3	Signal transduction histidine kinase with PAS and REC domains
SIVHX_1415	2	2	2	2	2	106	63.5	184.5	165	129.8	MenB; Naphthoate synthase
SIVHX_0816	2	2	2	2	2	113	122	111	167	128.3	PolB; DNA polymerase
SIVHX_2178	2	2	2	2	2	147.5	25.5	188	127.5	122.1	RimL; Acetyltransferase, RimL family
SIVHX_1113	2	2	2	1	2	15	17.5	294	162.5	122.3	VirB11; ATPase involved in archaellum/pili biosynthesis
SIVHX_2878	2	2	2	2	2	82.5	115.5	64.5	166	107.1	PdhB; Pyruvate/2-oxoglutarate dehydrogenase complex, dehydrogenase (E1) component, beta subunit
SIVHX_1552	2	2	2	0	2	161.5	122	0	17.5	75.3	SsuA; ABC-type sulfonate transport system, periplasmic component
SIVHX_2546	2	2	2	2	2	76.5	89	113.5	0	69.8	Uncharacterized protein
SIVHX_1691	2	2	2	2	2	79.5	132	0	90	75.4	GTP1; Ribosome-binding ATPase YchF, GTP1/OBG family
SIVHX_3054	2	2	2	2	2	129	42	0	48	54.8	AceB; Malate synthase, Citrate lyase beta subunit
SIVHX_1290	2	2	2	2	2	78	19	0	130.5	56.9	Uncharacterized protein
SIVHX_1736	2	2	2	2	2	46	60	16.5	89	52.9	Predicted component of the ribosome quality control (RQC) complex, YloA/Tae2 family
SIVHX_1911	2	2	2	1	2	85	12.5	0	104	50.4	Gfo/Idh/MocA family oxidoreductase
SIVHX_0081	2	2	2	2	2	30.5	11.5	0	187	57.3	AmtB; Ammonium transporter
SIVHX_0464	2	2	2	2	2	37	9	0	0	11.5	S-layer protein
SIVHX_1869	2	2	2	2	2	24.5	21	0	64	27.4	FixC; Electron transfer flavoprotein-quinone oxidoreductase
SIVHX_3004	2	2	2	1	2	29.5	26	64	11	32.6	Aor; Aldehyde:ferredoxin oxidoreductase
SIVHX_4151	2	2	2	2	2	33.5	18	0	0	12.9	PstS; ABC-type phosphate transport system, periplasmic component
SIVHX_0572	2	2	2	2	2	11	7	0	21.5	9.9	DNA-binding protein
SIVHX_2829	2	2	2	2	2	3	0	26.5	0	7.4	DppF; ABC-type dipeptide/oligopeptide/nickel transport system, ATPase component
SIVHX_2833	2	2	2	2	2	0	4.5	21	0	6.4	DppA; ABC-type dipeptide/oligopeptide/nickel transport system, periplasmic component
SIVHX_1169	2	2	2	2	2	5.5	7	0	16.5	7.3	CheF; Chemotaxis protein CheF
SIVHX_2707	2	2	2	2	2	7.5	9	0	0	4.1	NirA; Ferredoxin-nitrite reductase

*Numbers of GRGAaG sites within the gene bodies methylated at least in one experiment;

†Numbers of GRGAaG sites methylated in the experiments I, II, III and IV;

‡RPKM (reads per kilo base per million mapped reads) values of gene expression in the experiments I, II, III and IV.
